# Research priorities in gambling: Findings of a large-scale expert study

**DOI:** 10.1556/2006.2025.00072

**Published:** 2025-09-30

**Authors:** Andrea Czakó, Marc N. Potenza, David C. Hodgins, Shu M. Yu, Anise M. S. Wu, Susana Jiménez-Murcia, Henrietta Bowden-Jones, Daniel King, Joël Billieux, Beáta Bőthe, Dan J. Stein, Gambling Research Priority Setting Consortium, Zsolt Demetrovics, Gambling Research Priority Setting Consortium, Brett Abarbanel, Lucy Albertella, Jean-Marc Alexandre, Youssef Allami, Matteo Aloi, Laura Angioletti, Wen L. Anthony, Núria Aragay, Cecilia Åslund, Marc Auriacombe, Larry O. Awo, Stéphanie Baggio, Michael F. Baigent, Iris M. Balodis, Servane Barrault, Rafał P. Bartczuk, Malcolm Battersby, Maria E. Bellringer, Anne H Berman, Mónica Bernaldo-De-Quirós, Roshan Bhad, Alexander Blaszczynski, Marilisa Boffo, Céline Bonnaire, Marco Bortolato, Stephane Bouchard, Amy E. Bouchard, Matthias Brand, Helen Breen, Tim B. Brosowski, Matthew Browne, Tony W. Buchanan, Ursula G. Buchner, Gerhard Bühringer, Alessandra Buja, Natale Canale, Rene Carbonneau, Sari Castrén, Maris Catania, Gaëlle Challet-Bouju, Heather A. Chapman, Emeline Chauchard, Juliet Honglei Chen, Samwook Choi, Jung-Seok Choi, Mariano Chóliz, Darren R. Christensen, Jenny Cisneros Örnberg, Luke Clark, Irene Cogliati Dezza, David Columb, Olivier Corbeil, Aurélien Cornil, Pinhas Dannon, Christal N. Davis, Paul Delfabbro, Jeffrey L. Derevensky, Gaëtan Devos, Mike J. Dixon, Maria Anna Donati, Nicki A. Dowling, Magali Dufour, Simon Dymond, Enrique Echeburúa, Boris Egloff, Jennifer D. Ellis, Tara Elton-Marshall, Ana Estévez, Repairer Etuk, Shirley Fecteau, Fernando Fernández-Aranda, Naomi A. Fineberg, Gabriele Fischer, Mal Flack, David Forsström, Rebecca G. Fortgang, Ingmar H. A. Franken, Fabio Frisone, Johannes Fuss, Sally M. Gainsbury, Belle Gavriel-Fried, Anna M. Giannini, Montserrat Gómez García, Joaquín González-Cabrera, Adam S. Goodie, Alessio Gori, Anna E. Goudriaan, Marie Grall-Bronnec, Roser Granero, Mark D. Griffiths, Joshua B. Grubbs, Tara Hahmann, Anders Håkansson, Brian J. Hall, Juho Hamari, Wei Hao, Tobias Hayer, Robert M. Heirene, Nerilee Hing, Niklas Hopfgartner, Tristan J. Hynes, Richard J. E. James, Paula Jauregui, Emilien Jeannot, Andrew S. Kayser, Yasser Khazaal, Hyoun S. Kim, Serena King, Keiji Kobara, Komathi Kolandai, Toula Kourgiantakis, Ildikó Kovács, Shane W. Kraus, Ludwig Kraus, Søren Kristiansen, Daria J. Kuss, Jason Landon, Debi A. LaPlante, Bernard Le Foll, David M. Ledgerwood, Bonnie K. Lee, Bernadeta Lelonek-Kuleta, En Li, Kalle Lind, Philip Lindner, Jakob Linnet, Suzanne Lischer, Joanne Lloyd, Helen M. Lloyd, Christine Lochner, Hibai Lopez-Gonzalez, Valentina Lorenzetti, Eric R. Louderback, Johanna K. Loy, Amandine Luquiens, Joseph Macey, Juan M. Machimbarrena, Laura Macía Guerrero, Núria Mallorquí-Bagué, Viktor Månsson, Loredana A. Marchica, Emanuela Mari, Simon Marmet, Giovanni Martinotti, Flora I. Matheson, Sachio Matsushita, André J. McDonald, Daniel S. Mcgrath, Jose Menchon, Stephanie S. Merkouris, Gemma Mestre-Bach, Devin J. Mills, Lorenzo Moccia, Olof Molander, Sabrina Molinaro, Pedro Morgado, Franziska Motka, Viktor Mravcik, Lucero Munguía, W. Spencer Murch, Juan F. Navas, Sarah E. Nelson, Laura L. Nicklin, Giovanna Nigro, Anders Nilsson, Xavier Noel, Lia Nower, Colin O’Gara, Jane E. Oakes, Ugo Pace, Stefano Pallanti, Ståle Pallesen, Arpit Parmar, Jonathan Parke, Alberto Parrado-González, Alessia Passanisi, Raimondo M. Pavarin, José C. Perales, Jan Peters, Rory A. Pfund, Kahlil S. Philander, Fulvia Prever, Alessandro Quaglieri, Jonas Rafi, Carla J. Rash, Vijay Rawat, Jérémie Richard, Neven Ricijas, Amanda Roberts, Simone N. Rodda, Jim Rogers, Guyonne Rogier, Sara Rolando, Nina Romanczuk-Seiferth, Don Ross, Hans-Jürgen Rumpf, Gillian E. H. Russell, Alex M. T. Russell, Paul Sacco, Dominic Sagoe, Anne H. Salonen, Eva Samuelsson, James L. Sanders, John B. Saunders, Michael P. Schaub, Mauro Schiavella, Adriano Schimmenti, Casper Schmidt, Guillaume Sescousse, Serge Sévigny, Howard J. Shaffer, Steve Sharman, Jing Shi, Steven D. Shirk, Lucia Sideli, Olivier Simon, Pawel Sleczka, Ryuhei So, Rhys M. G. Stevens, Trevor Steward, Mythily Subramaniam, Thomas B. Swanton, André Syvertsen, Nassim Tabri, Hidehiko Takahashi, Stefano Tamburin, So Kum C. Tang, Éric R. Thériault, Shane A. Thomas, Kristine R. Thomsen, Alexander Tomei, Kwok Kit Tong, Joan Trujols, Samson Tse, Kosuke Tsurumi, Catherine Tulloch, Richard Tunney, Eduardo Valenciano-Mendoza, Mark van der Maas, Thilo Van Eimeren, William Van Gordon, Ruth J. Van Holst, Tim Van Timmeren, Patrizia Velotti, Cristina Vintró-Alcaraz, Rachel Volberg, Kristin M. von Ranson, Kathrin Weidacker, James P. Whelan, Seth Whiting, Łukasz Wieczorek, Robert Williams, Ken C. Winters, Michael J. A. Wohl, Patrick D. Worhunsky, Leon Y. Xiao, Kengo Yokomitsu, Murat Yucel, Martin H. Zack, Meng Xuan Zhang, Alexander Zink, Francesca Zoratto

**Affiliations:** 1 Centre of Excellence in Responsible Gaming, University of Gibraltar, Gibraltar, Gibraltar; 2 Institute of Psychology, ELTE Eötvös Loránd University, Budapest, Hungary; 3 Department of Psychiatry, Yale University School of Medicine, New Haven, CT, USA; 4 Child Study Center, Yale University School of Medicine, New Haven, CT, USA; 5 Connecticut Mental Health Center, New Haven, CT, USA; 6 Connecticut Council on Problem Gambling, Wethersfield, CT, USA; 7 Department of Neuroscience, Yale University, New Haven, CT, USA; 8 Wu Tsai Institute, Yale University, New Haven, CT, USA; 9 Department of Psychology, University of Calgary, Calgary, Canada; 10 Department of Psychology, Faculty of Social Sciences, University of Macau, Macao, China; 11 Centre for Cognitive and Brain Sciences, Institute of Collaborative Innovation, University of Macau, Macao, China; 12 Clinical Psychology Department, Bellvitge University Hospital- Bellvitge Biomedical Research Institute (IDIBELL), Barcelona, Spain; 13 Ciber Physiopathology of Obesity and Nutrition (CIBERObn), Instituto de Salud Carlos III, Barcelona, Spain; 14 Psychoneurobiology of Eating and Addictive Behaviors Group, Neurosciences Programme, Bellvitge Biomedical Research Institute (IDIBELL), Barcelona, Spain; 15 Department of Clinical Sciences, School of Medicine and Health Sciences, University of Barcelona, Barcelona, Spain; 16 Department of Psychiatry, University of Cambridge, UK; 17 National Problem Gambling Clinic & National Centre for Gaming Disorders, London, UK; 18 Department of Brain Sciences, University College London, London, UK; 19 School of Psychology, The University of Adelaide, Australia; 20 Flinders University Institute for Mental Health and Wellbeing, College of Education, Psychology and Social Work, Flinders University, Bedford Park, SA, Australia; 21 Institute of Psychology, University of Lausanne, Lausanne, Switzerland; 22 Centre for Excessive Gambling, Addiction Medicine, Lausanne University Hospitals (CHUV), Lausanne, Switzerland; 23 Département de Psychologie, Université de Montréal, Montréal, Canada; 24 SAMRC Unit on Risk & Resilience in Mental Disorders, Dept of Psychiatry & Neuroscience Institute, University of Cape Town, South Africa

**Keywords:** gambling, gambling disorder, research priorities, addictive behavior, compulsive behavior, impulsive behavior, behavioral addiction, expert study, policy, treatment, intervention, prevention

## Abstract

**Objective:**

While gambling is a growing public health concern, research resources are limited, and no guidance is available to prioritise research. This study aimed to identify priorities for gambling research on a global scale using a systematic, transparent, and democratic methodology to inform researchers and other stakeholders.

**Methods:**

Leading gambling researchers were invited to list gambling-related research questions that can contribute to strengthening evidence-based policy, prevention, and effective early intervention and treatment of problem gambling. Suggestions were consolidated into research options and evaluated against six criteria (Answerability, Feasibility, Effectiveness, Impact on equity and an additional two based on the category of research options: Novelty and Relevance for description-type, Potential for burden reduction and Deliverability for intervention-related options). Stakeholders ( *n* = 14) assigned relative weights to each criterion, and options were ranked according to their weighted research priority scores.

**Results:**

With input from 46.9% of eligible researchers ( *n* = 307) from 35 countries, 1,361 questions were consolidated into 102 options. Evaluations showed strong agreement between experts, and the top 25 priorities were identified. The results highlight the need for further knowledge about the epidemiology, etiology, and consequences of problem gambling. Top-priority topics indicate the importance of focusing on vulnerable and minority groups, youth, significant others, technological innovations, advertisements, the convergence of gaming and gambling, and co-occurring conditions. Evaluating and tailoring existing measures were prioritised more highly than new interventions, and identifying factors underlying treatment seeking, drop-out and relapse was also considered a priority.

**Conclusions:**

This initiative successfully involved the global research community in identifying gambling research priorities. The results provide information for researchers and other stakeholders for future projects and funding.

## Introduction

Gambling has experienced considerable expansion in the past decades and is now a legal activity in 80% of countries ( Ukhova, Marionneau, Nikkinen, & Wardle, 2024). Gambling is generally considered a leisure activity, but it has also been recognised to have an addictive potential and negatively affect many people. Adverse consequences include financial, emotional, relational, and other harms, decreased work performance, and criminality ( Langham et al., 2015). While a minority of people who gamble experience clinically significant impairments recognised as gambling disorder ( American Psychiatric Association, 2022; World Health Organization, 2019), harms from gambling are also experienced by those who do not meet the diagnostic criteria of gambling disorder ( Browne & Rockloff, 2018) and by significant others of people who gamble ( Langham et al., 2015). Gambling also generates substantial economic burdens on societies, with the total burden approaching the levels of harm of major depressive or alcohol use disorders ( Browne et al., 2016). The international prevalence of problem gambling is 1.41% in the adult population, according to a recent meta-analysis ( Tran et al., 2024), although systematic reviews report variability in prevalence estimates related to methodological, geographical, and cultural differences ( Gjoneska et al., 2025).

Despite personal, familial, and societal harms, gambling has often been neglected as a public health issue ( Wardle, Degenhardt, Ceschia, & Saxena, 2021) and only recently started to be recognised as a serious public health concern ( Ukhova et al., 2024) that requires evidence-based strategies to tackle related harms and reduce their impact, increased levels of research and action at national and international levels ( Wardle et al., 2024). Gambling-related research has also increased in the past decades: the number of papers published in 2023 featuring the search term “gambl*” in the title or abstract in the Web of Science database was 759, more than three times higher than in 2003 ( *n* = 217) and eleven times higher than in 1993 ( *n* = 66). These manuscripts examine different aspects of gambling, including non-problematic, problematic, and disordered gambling and from different disciplinary perspectives, from genetics and neuroscience to psychological and social features, treatment, and policy issues. This rise indicates a continued need for empirical investigation towards understanding the development and maintenance of disordered gambling, how the related personal, familial, social, and economic burdens may be reduced, and how evidence-based prevention, treatment, and policy measures may be implemented.

As financial and human capacities for gambling research are limited, it is important to focus on the most pressing questions and establish priorities to properly inform stakeholders in gambling-related domains, including research communities, policymakers, and funding organisations. Nevertheless, few comprehensive initiatives have been undertaken and none have used systematic methodologies to consider gambling-related research at the international level. The consensus view of the National UK Research Network for Behavioural Addictions aimed to identify key gambling research priorities focusing solely on the United Kingdom ( Bowden-Jones et al., 2022). In an earlier initiative, a team of international experts identified knowledge gaps and created a list of future research areas as a secondary goal linked to their comprehensive framework of harmful gambling ( Abbott et al., 2018). Others applied a broader thematic focus that included gambling, such as the problematic use of the internet ( Fineberg et al., 2018) or addiction research in general ( West et al., 2019).

To fill this gap, a Research Priority Setting in Gambling Project Core Group (PCG) was created to identify research priorities (i) specifically in the gambling field, (ii) on a global level, and (iii) applying a systematic methodological approach.

## Methods

The exercise adapted the Child Health and Nutrition Research Initiative (CHNRI) methodology, a transparent and democratic method developed to assist decision-making and consensus development in child health and nutrition ( Rudan, Gibson, et al., 2008), and used later in various health domains ( Rudan et al., 2017). Similar to most CHNRI exercises, the original methodology was amended to best suit the objectives.

The PCG, comprised of eight researchers from leading gambling research institutes and representing diversity in sex, geography, and research focus, defined the context, designed the methodology and conducted the project.

A comprehensive perspective was adopted when delineating the context for the research priorities, with an overarching aim to identify those gambling-related research areas that should be prioritized to strengthen existing evidence-based policy, prevention, and effective early intervention and treatment of problem gambling and gambling-related harms. The population of interest, those whose problems were aimed to be addressed, was defined on a global level and included all who have ever experienced or are at risk of experiencing any gambling-related harm, their families, affected others, communities, and societies in general. The timeframe for research priorities was the next five years.

**Fig. 1.**
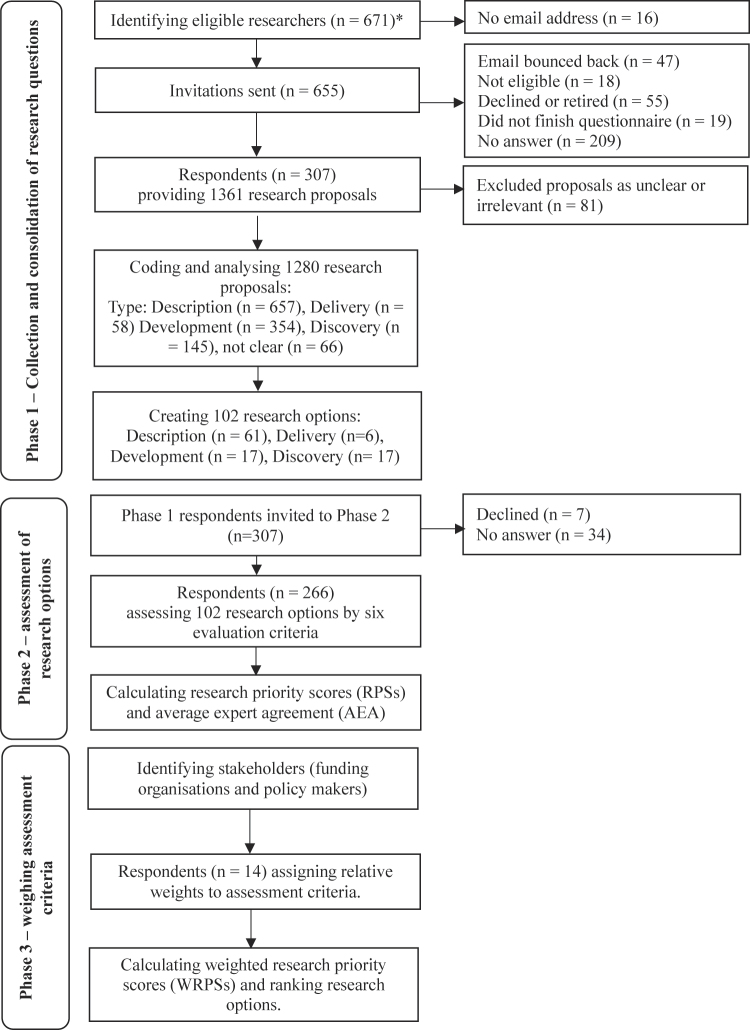
Overview of the research priority setting process *PCG members were excluded from the list of participants.

The project was reviewed for ethical acceptability, approved by the University of Gibraltar *,* and preregistered on the Open Science Framework ( https://osf.io/abn3e) *.* It was conducted in three phases (see Fig. 1) and involved a diverse group of researchers and other stakeholders worldwide.

Phase 1 aimed to identify relevant research questions with the help of experts involved in gambling research. The inclusion criteria were being the first, last, or corresponding author of at least two gambling-related scientific papers. To identify eligible experts, a systematic database search was conducted in Web of Science using the keyword “Gambl*” within the title or abstract of papers published between 2017 and 2021. Experts were invited to complete an online survey and list 3 to 6 distinct research questions/avenues that they believed to be the most important to address in the next five years and provide information regarding their demographics, such as gender, geographical location and highest level of education, the number and types of affiliations, area of expertise, self-reported level of expertise in gambling in general and in gambling research, and scientific outputs such as publications and successful gambling-related research grants. Conflicts of interest were collected and transparently reported. Researchers contributing to all phases were invited to have group authorship provided they read, commented on and acknowledged the results and the manuscript. Phase 1 data were collected between August 2022 and February 2023.

The collected research suggestions were coded using ATLAS.ti Web ( ATLAS.ti Scientific Software Development GmbH, 2023). Irrelevant and unclear responses were removed, and duplicates, redundancies, and overlaps were combined by two authors (AC & SMY). Following the CHNRI framework that organises proposed research topics by their depth into (1) broad research domains, (2) research avenues, (3) research options, and (4) specific research questions ( Rudan, Chopra, et al., 2008), the depth of the final list was set at the level of *research option* corresponding to research programs for which several research projects with different methodological backgrounds could be designed to answer multiple specific research questions. To reach a uniform level, suggestions focusing on narrow, specific questions were incorporated into broader topics. The language was standardised to help the scoring, e.g., *problem gambling* was used as a broader term for all levels of problematic gambling, including gambling disorder, while the term *gambling disorder* specifically referred to the mental health disorder defined in the Diagnostic and Statistical Manual of Mental Disorders of the American Psychiatric Association (DSM-5) and the International Classification of Diseases of WHO (ICD-11).

The final list was sorted into (1) description-type research aiming to understand problem gambling, investigate prevalence, underlying causes, consequences, and burdens, (2) delivery-type research aiming to improve the delivery and accessibility of available measures and interventions, (3) development-type research aiming to evaluate or improve the effectiveness and sustainability of measures and interventions, and (4) discovery-type research aiming to innovate and develop new interventions, preventions, treatments, or policies.

For the assessment of the research options, the PCG adopted eight criteria (see Table 1). As a modification of the CHNRI methodology, two sets of six criteria were used depending on the assessed question type. The first four criteria, Answerability, Feasibility, Effectiveness, and Impact on equity, were applied to all research options, while the last two criteria varied depending on the question type. The additional two criteria for *description-type research options* were Novelty and Relevance, while for the *intervention-focused delivery, development, and discovery-type options*, these were Potential for burden reduction and Deliverability of the intervention.

**Table 1.** Criteria for assessing the research options

Criterion	Description	Relative weight* for the assessment of description-type questions	Relative weight* for the assessment of intervention-focused questions
Answerability	Can a study/studies be designed to answer the research question and to achieve the proposed aims of the research?	1.05	0.91
Feasibility	Are the necessary resources, conditions, and capacities available to conduct such research within the 5-year timeframe?	0.93	0.81
Effectiveness	Is the research likely to lead directly or indirectly to the development or improvement of effective measures (e.g., policies, interventions, treatment) within the 5-year timeframe?	1.12	1.18
Impact on equity	Does the proposed research have the potential to contribute to equity in disease burden distribution, for example, by increasing the availability of treatment and prevention for specific groups?	0.92	0.86
Novelty	Is the research likely to generate novel results that significantly add to our current knowledge?	0.69	NA
Relevance	Will the proposed research contribute to addressing knowledge gaps that are highly relevant to the overall understanding of problem gambling?	1.29	NA
Potential for burden reduction	Does this research have the potential to contribute significantly to *reducing the burden of problem gambling* on people who gamble, significant others, and society?	NA	1.15
Deliverability of intervention	Would there be *sufficient available resources* (infrastructure, human capacity) and support from relevant stakeholders *to successfully implement the developed measures* (e.g., intervention, policy) or the improvement of these measures?	NA	1.09

*The relative weights were assigned by stakeholders in Phase 3.

NA = Not applicable.

Phase 2 data were collected between May and September 2023. Phase 1 participants were invited to rate each option against six criteria considering a general, global perspective (yes (1 score), no (0 score), maybe (0.5 score), or I don't know (missing)). These ratings were averaged for each criterion and for an overall Research Priority Score (RPS).

The level of average expert agreement (AEA) was determined for each research option by dividing the number of most frequent answers by the number of responses for each criterion and calculating an average of these criteria-level agreement scores. “Maybe” answers, not representing a definite opinion, were not included in the calculation.

To include the perspectives of gambling research beneficiaries, a broader range of stakeholders, including organisations providing funding for gambling-related research, gambling regulators, and policymaking bodies were involved in Phase 3. This phase aimed to determine the relative importance of each evaluation criterion (i.e., weighted research priority scores [WRPSs]). The list of invited organisations was compiled to be geographically diverse. Stakeholders were contacted through email and asked to complete an anonymous online survey, and distributing 100 scores among the six evaluation criteria in both sets of criteria. The relative weight of each criterion was calculated by dividing the mean value of the scores by 16.7 (100/6). Weights were calculated separately for the two sets of criteria. The research options were ranked according to their WRPS values, creating two ranks (i.e., description-type and intervention-focused) for research options. Analyses were performed using IBM SPSS 27 and Microsoft Excel.

## Results

### Phase 1

In Phase 1, a total of 671 eligible researchers were identified. After three rounds of reminder emails, 46.9% of the invited experts completed the questionnaire ( *n* = 307, male = 58.6%, *M*_age_ = 46.1, SD = 11.38; see Table 2). Participants were from 35 countries on 6 continents, most from Europe (54.2%) and North America (27.5%). Almost all (95.8%) were affiliated with universities or academic research institutes, and 17.6% with healthcare service provision. Most participants (86.6%) had a PhD degree. Regarding expertise, 71.3% listed psychology, 30.6% public health, 29.6% psychiatry, 22.5% neurosciences, and 20.8% epidemiology (see the full list of expertise in Table 2). The average level of self-reported expertise as a gambling researcher was 3.8 (SD = 0.87) (1 = very low to 5 = very high), while the average length for gambling-related research involvement was 12.2 years (SD = 7.11). One-third published more than 20 gambling-related peer-reviewed manuscripts, and most had been involved in gambling-related research grants as a collaborator (63.9%) or principal investigator (55.3%; see Tables 2 and 3).

**Table 2.** Demographic characteristics and expertise of Phase 1 and Phase 2 respondents

	Phase 1		Phase 2	
	*n*	%	*n*	%
**Gender**	***n* = 304 **		***n* = 263 **	
Male	178	58.6%	157	59.7%
Female	126	41.4%	106	40.3%
**Geographical location**	***n* = 306 **		***n* = 265 **	
Europe	166	54.2%	145	54.7%
North America	84	27.5%	67	25.3%
South America	1	0.3%	1	0.4%
Asia	21	6.9%	21	7.9%
Africa	4	1.3%	3	1.1%
Oceania	30	9.8%	28	10.6%
**Racial identity**	***n* = 287 **		***n* = 249 **	
Indigenous	1	0.3%	0	0.0%
Black/African	4	1.4%	3	1.2%
East Asian	22	7.7%	21	8.4%
South Asian	6	2.1%	5	2.0%
Hispanic	6	2.1%	5	2.0%
Middle Eastern	3	1.0%	2	0.8%
White/Caucasian	241	84.0%	210	84.3%
Mixed	3	1.0%	2	0.8%
Other	1	0.3%	1	0.4%
**Highest degree of education**	***n* = 307 **		***n* = 266 **	
Bachelor's degree	2	0.7%	2	0.8%
Master's degree	26	8.5%	25	9.4%
PhD or equivalent	266	86.6%	228	85.7%
Other	13	4.2%	11	4.1%
**Number of affiliations**	***n* = 307 **		***n* = 266 **	
1	176	57.3%	150	56.4%
2	94	30.6%	80	30.1%
3	31	10.1%	30	11.3%
4	6	2.0%	6	2.3%
**Type of affiliation (multiple selection was possible)**	***n* = 306 **		***n* = 266 **	
Academic (university or research institute)	293	95.8%	256	96.2%
Governmental Administration Body	6	2.0%	4	1.5%
Company/industry	6	2.0%	5	1.9%
Counselling, education, prevention institute/centre	1	0.3%	0	0.0%
Health care service provider	54	17.6%	50	18.8%
Other	10	3.3%	10	3.8%
**Area of expertise (multiple selection)**	***n* = 307 **		***n* = 266 **	
Biology	10	3.3%	9	3.4%
Business and Economics	11	3.6%	11	4.1%
Computer Science and Mathematics	7	2.3%	7	2.6%
Cultural anthropology	10	3.3%	8	3.0%
Education Science	4	1.3%	4	1.5%
Epidemiology	64	20.8%	56	21.1%
Law/Legal Studies	10	3.3%	7	2.6%
Medicine and Health	52	16.9%	46	17.3%
Methodology	33	10.7%	29	10.9%
Neurosciences	69	22.5%	60	22.6%
Philosophy and humanities	5	1.6%	4	1.5%
Political science	8	2.6%	6	2.3%
Psychiatry	91	29.6%	80	30.1%
Psychology	219	71.3%	193	72.6%
Public Health	94	30.6%	81	30.5%
Social Sciences	58	18.9%	49	18.4%
Sociology	20	6.5%	17	6.4%
Statistics/data science	33	10.7%	29	10.9%
Other	18	5.9%	15	5.6%
**Number of gambling related papers published**	***n* = 299 **		***n* = 260 **	
Less than 5	75	25.1%	64	24.6%
Between 6 and 10	63	21.1%	56	21.5%
Between 11 and 20	60	20.1%	52	20.0%
More than 20	101	33.8%	88	33.8%
**Number of gambling related papers published as a lead author**	***n* = 296 **		***n* = 258 **	
Less than 5	133	44.9%	111	43.0%
Between 6 and 10	65	22.0%	57	22.1%
Between 11 and 20	47	15.9%	44	17.1%
More than 20	51	17.2%	46	17.8%
**Number of successful gambling-related research grants as a principal investigator in the past 5 years**	***n* = 295 **		***n* = 255 **	
0	132	44.7%	111	43.5%
1	62	21.0%	55	21.6%
2	37	12.5%	29	11.4%
3	16	5.4%	15	5.9%
4	9	3.1%	7	2.7%
5	12	4.1%	12	4.7%
≥6	27	9.2%	26	10.2%
**Number of successful gambling-related research grants as a collaborator in the past 5 years**	***n* = 285 **		***n* = 250 **	
0	103	36.1%	91	36.4%
1	59	20.7%	47	18.8%
2	41	14.4%	37	14.8%
3	28	9.8%	27	10.8%
4	11	3.9%	10	4.0%
5	7	2.5%	6	2.4%
≥6	36	12.6%	32	12.8%

**Table 3.** Gambling-related expertise of Phase 1 and Phase 2 respondents

	Phase 1					Phase 2				
	*N*	Min	Max	Mean	SD	*N*	Min	Max	Mean	SD
Proportion of time spent with the different gambling related professional activities										
Research	304	0	100	69.7	27.37	263	0	100	70.1	27.11
Education	304	0	70	10.8	13.21	263	0	70	10.8	13.20
Prevention	304	0	100	4.2	9.56	263	0	50	3.9	8.04
Clinical/treatment work	304	0	90	9.4	18.06	263	0	90	9.6	18.55
Policy related work	304	0	35	3.5	6.73	263	0	35	3.5	6.73
Other gambling related work	304	0	100	2.4	11.62	263	0	100	2.1	10.41
Level of expertise within the field of gambling as a whole (1 = very low, 5 = very high)	304	1	5	3.7	0.94	263	2	5	3.8	0.92
Level of expertise as a gambling researcher (1 = very low, 5 = very high)	304	1	5	3.8	0.87	263	1	5	3.9	0.85
Number of years involved in gambling research	307	1	42	12.2	7.11	266	3	42	12.2	7.14

Seventy-three participants (23.9%) confirmed having had a relationship with the gambling industry in the past five years. The nature of this relationship varied from consultancy to receiving data and research funding. Further information is provided in the conflict-of-interest statements ( Supplementary material).

The participants listed 1,361 research questions, of which 81 were excluded as being unclear or irrelevant. Based on PCG analysis and discussions, the proposals were consolidated into 102 research options: 61 (59.8%) description-type and 41 (40.2%) intervention-focused.

### Phase 2

Among the 307 invited experts, 86.6% ( *n* = 266) participated in Phase 2 and assessed the 102 research options. Their demographic characteristics and expertise were comparable to those in Phase 1 ( Table 2). RPSs of the research options ranged from 0.585 to 0.839, while AEA levels ranged from 66.2% to 95.9% ( Appendix).

### Phase 3

Representatives of 14 stakeholder organisations: eight from Europe, three from North America, and three from Australia, assessed the relative importance of the evaluation criteria. Weights assigned to the evaluation criteria are presented in Table 1.

WRPSs of the research options ranged from 0.584 to 0.848. Table 4 presents the top quarter of research options in both groups, including 15 from the description-type research options and 10 from the intervention-focused research options. AEA among these 25 options ranged from 88.7% to 95.9%.

**Table 4.** The 25 highest-ranking research priorities for gambling (WRPS = Weighted Research Priority Score, AEA = average expert agreement)

	Research option	Type of option	Theme	WRPS	AEA	Rank within question type	Overall rank
A Description-type	Investigating factors related to treatment outcomes for people with gambling disorder	Description	Treatment	0.848	94.9%	1	1
	Studying the epidemiology of problem gambling in vulnerable populations (e.g., underrepresented minority groups, individuals with mental disorders or brain injuries, low-income household members, homeless individuals)	Description	Epidemiology	0.845	94.8%	2	2
	Epidemiological research on gambling among adolescents and young adults	Description	Epidemiology	0.819	91.9%	3	4
	Epidemiological research on new forms of gambling	Description	Epidemiology	0.817	94.4%	4	5
	Studying the nature and harms related to newer forms of gambling and gambling-like activities (e.g., in-play betting, fantasy sports, cryptocasinos, esports betting, virtual reality gambling)	Description	Consequence	0.816	95.0%	5	6
	Investigating the role of gambling-focused advertising (including sponsorship, streaming platforms, online influencers) in problem gambling among youth	Description	Etiology	0.810	94.6%	6	7
	Investigating risk and protective factors of gambling problems among adolescents and young adults	Description	Etiology	0.806	92.5%	7	10
	Investigating the treatment needs of minority populations (ethnic, cultural, linguistic, gender, sexual, immigrant, etc.)	Description	Treatment	0.799	95.1%	8	11
	Assessing the impact of gambling and related harms in the case of significant others (children, partners, other family members) and investigating strategies of coping	Description	Consequence	0.796	93.3%	9	13
	Investigating the gambling behaviour and problem gambling in minority groups (ethnic, cultural, linguistic, gender, sexual, immigrant, etc.)	Description	Etiology	0.789	93.8%	10	15
	Cross-cultural epidemiological studies of problem gambling (e.g., across time, different jurisdictions and countries with different economic conditions)	Description	Epidemiology	0.787	92.3%	11	17
	Epidemiological research on the co-occurrence of problem gambling and non-gambling somatic, mental health and addictive disorders	Description	Epidemiology/comorbidity	0.784	90.0%	12	19
	Reaching a scientific consensus on the definition and empirically based measures of at-risk gambling and problem gambling	Description	Taxonomy	0.781	88.7%	13	20
	Studying the longitudinal relationship between gambling-like activities (e.g., loot boxes, social casino games), gambling engagement and problem gambling	Description	Etiology	0.776	89.7%	14	22
	Studying the individual and environmental factors of relapse in problem gambling	Description	Treatment	0.773	90.6%	15	24
B Intervention-focused (delivery, development and discovery type)	Investigating the effectiveness of mobile/online tools that increase the accessibility of problem gambling interventions	Delivery	Treatment	0.830	95.8%	1	3
	Identifying factors that hinder treatment-seeking for problem gambling	Delivery	Treatment	0.809	95.9%	2	8
	Evaluating the effectiveness of existing psychosocial treatments for gambling disorder	Development	Treatment	0.807	94.3%	3	9
	Identifying factors behind dropping out of problem gambling treatment	Delivery	Treatment	0.798	95.8%	4	12
	Evaluating the effectiveness of existing online and mobile gambling interventions for at-risk and problem gambling	Development	Prevention	0.792	93.7%	5	14
	Evaluating the effectiveness of existing gambling problem prevention programs for adolescents and young adults	Development	Prevention	0.788	95.1%	6	16
	Formulation of evidence-based recommendations for the regulation of gambling-related advertisements	Discovery	Policy	0.785	93.7%	7	18
	Evaluating the effectiveness of existing treatments for gambling disorder co-occurring with other addictive or mental health disorders	Development	Treatment	0.778	93.9%	8	21
	Development of evidence-based interventions to prevent relapse	Discovery	Prevention	0.776	91.9%	9	23
	Tailoring evidence-based treatments for subgroups of people with gambling disorder (e.g., youth, adolescents, older adults, women, minorities)	Development	Treatment	0.773	93.5%	10	25

### Description-type research option priorities

*Epidemiological themes* focusing on different populations and gambling forms were the most prominent, including the epidemiology of problem gambling in vulnerable populations, adolescents and youth, and epidemiological research on emerging forms of gambling and on the co-occurrence of problem gambling and other disorders (A2, A3, A4, A11, A12).

The second most prominent theme was *etiological research* (A6, A7, A10, A14) investigating the role of gambling-focused advertising among youth, risk and protective factors of gambling problems among adolescents and young adults, gambling in minority groups, and longitudinal relationships between gambling-like activities and gambling.

Three research options focused on treatment-related topics. Investigating *factors related to treatment outcomes* was ranked first (A1), with an AEA of 94.9%. Other topics included the treatment needs of minority populations (A8) and relapse in problem gambling (A15).

Two options focused on the *harms and negative consequences of gambling (A5, A9)*: studying harms related to newer forms of gambling and gambling-like activities and harms experienced by significant others such as children, partners, and other family members and their strategies of coping. One *taxonomy-themed* research option suggested reaching a consensus on the definition and empirically based measures of at-risk and problem gambling (A13).

There were four options among the top fifteen according to the two group-specific criteria (i.e.: Relevance and Novelty) that were not included in the overall fifteen due to scoring relatively low on the Answerability and Feasibility criteria. These included the investigation of gambling-related policymaking and barriers to meaningful changes, establishing globally harmonised psychometric tools for cross-cultural research, assessing the social cost and public health impact of problem gambling across countries, and differentiating between harm from problem gambling and harm from co-occurring conditions (A18, A19, A22, A37).

### Intervention-focused research option priorities

Five of the top ten options were development-type, three were delivery-type, and two were discovery-type. Six focused on treatment, three on prevention, and one on policy.

*Treatment-themed options included* investigating the effectiveness of mobile/online tools, psychosocial treatments, and treatments for gambling disorder co-occurring with other disorders, identifying factors that hinder treatment-seeking and factors behind treatment drop-out and tailoring treatments for subgroups of people such as youth, older people, women, and minorities (B1, B2, B3, B4, B8, B10).

From the *three prevention-themed options*, the evaluation of youth prevention programs, online and mobile interventions for at-risk gambling, and the creation of interventions to prevent relapse were the emerging topics (B5, B6, B9). The *policy-themed research option* suggested formulating evidence-based recommendations for the regulation of gambling advertisements (B7).

## Discussion

This global priority-setting exercise identified the most pressing questions in gambling research through a well-defined process involving gambling researchers. More than half of the experts had led successful gambling related research grants as principal investigators in the past five years, and the majority has published more than five gambling related papers as a lead author.

Although the suggestions identified a wide range of topics, there was strong agreement regarding the most important research gaps. Several overlapping themes, objectives, populations, and methodological requirements emerged from the highly prioritised research options. The high proportion of descriptive research questions indicates that despite the increasing amount of gambling-related research over the past decades, there is a need to generate further *fundamental knowledge about the epidemiology, risk and protective factors of problem gambling and gambling-related harms*. This aligns with global research priorities set for other mental health disorders that include research on root causes, risk and protective factors ( Collins et al., 2011), and also with UK research priorities that include longitudinal research on the prevalence of disordered gambling and gambling-related harms ( Bowden-Jones et al., 2022). Although understanding the neurobiological basis of gambling disorder, which was one of the priorities set in the UK, was included in the listed research options, it did not emerge as a priority topic in this exercise.

The results highlight a need for an increased focus on *vulnerable populations* relating to ethnic, cultural, linguistic, gender, sexual, educational, and income factors. The prevalence of gambling problems in vulnerable groups, specific gambling-related harms and treatment needs require further exploration, especially as several of the above characteristics, including poor educational attainment and financial problems, were previously identified as risk factors for gambling disorder ( Moreira, Azeredo, & Dias, 2023). The few available studies conclude that certain minority groups appear more vulnerable to developing gambling disorder ( Okuda et al., 2016), tend to start to gamble and develop gambling problems at younger ages, and experience more negative consequences when diagnosed with gambling disorder ( Grant & Chamberlain, 2023). The lack of research investigating gambling in sexual and gender minorities has also been noted ( Gartner, Bickl, Härtl, Loy, & Häffner, 2022; Lee & Grubbs, 2023).

*Multiple specific concerns involve adolescents and young people*. They are at heightened risk of problem gambling, likely due to their emotional and cognitive immaturity and increased susceptibility to peer influences and advertisements ( Emond & Griffiths, 2020). According to the UK Gambling Commission report, 26% of teenagers have gambled for money within the past year, and 0.7% have experienced problem gambling ( Young People and Gambling, 2023). Future research should focus on understanding the determinants of youth gambling, how these change over time ( Calado, Alexandre, & Griffiths, 2017), and how health impacts and negative consequences might extend to adulthood ( Armitage, 2021).

Research related to *significant others* of those who gamble was also highlighted. Negative consequences of gambling impact close individuals ( Langham et al., 2015), including emotional, relational, and financial, health, and other harms, especially among former and present partners and family members ( Hing et al., 2022). As these harms are associated with substantial distress, exploring causalities and the development and nature of harms needs further investigation ( Tulloch, Browne, Hing, Rockloff, & Hilbrecht, 2023).

Two themes shifted the focus from those who gamble and experience gambling-related harms to the gambling industry. One was the *investigation of the risks and consequences of new technological innovations, emerging forms of gambling and gambling-like activities, including gambling-like elements of video games.* Features in the intersection of video games and gambling, such as loot-boxes, disproportionately affect youth, create challenges for families ( Király, Zhang, Demetrovics, & Browne, 2021), and may promote gambling harms ( Zendle & Cairns, 2018). Furthermore, emerging and rapidly changing technologies, including new devices, designs, personalised marketing strategies, and artificial intelligence, may increase the accessibility of gambling, create new risks and increase the ways people experience harm ( Swanton, Blaszczynski, Forlini, Starcevic, & Gainsbury, 2021). The other theme concerned research on *gambling-focused advertising,* especially in relation to youth vulnerability, and formulating evidence-based recommendations for regulations. Although research focusing on advertising increased in the past decade, the pace and range of methods and topics should be expanded ( Torrance et al., 2021).

Regarding research into prevention and treatment, the empirical *evaluation of the effectiveness of existing measures* was generally more highly prioritised than the creation of new interventions. This aligns with the UK research priorities, pressing the need to conduct randomised controlled trials on interventions and to investigate factors related to successful outcomes ( Bowden-Jones et al., 2022), in line with previously set global mental health research priorities ( Tomlinson, 2009). However, the range of measures to be evaluated needs to be determined, and a focus should be placed on methodologically rigorous, high-quality studies ( Brand et al., 2025).

Regarding the improvement of the accessibility and delivery of existing interventions, results highlight the importance of acknowledging the heterogeneity of people who gamble and *tailoring existing preventive and treatment measures* to the needs of different groups, including vulnerable groups, young people, women, and minorities. The *accessibility of available treatments* was also identified as an important research priority theme, including investigating the barriers to treatment-seeking and factors linked to dropping out. Despite the availability of various treatment services and self-help options, help-seeking among people with gambling problems remains low, with one-fifth or less seeking any help ( Bijker, Booth, Merkouris, Dowling, & Rodda, 2022), and four out of ten dropping out ( Pfund et al., 2021). Results suggest that individual and environmental factors related to *relapse* also need further exploration. Although there is a high rate of reoccurrence of gambling disorder after recovery, studies exploring predictive factors and long-term follow-up studies are scarce ( Grall-Bronnec et al., 2021).

The *co-occurrence of physical and mental health disorders* was another key theme, signalling a need for related epidemiological research and the evaluation of treatment effectiveness. Gambling disorder is frequently associated with co-occurring mental disorders, for example, substance use, mood, anxiety, and personality disorders ( Petry, Stinson, & Grant, 2005). However, there exists limited knowledge about the complex temporal and causal relationships between these different conditions and the underlying etiological factors ( Hartmann & Blaszczynski, 2018). Furthermore, as these comorbid mental disorders are associated with higher problem-gambling severity and poorer treatment success ( Wullinger, Bickl, Loy, Kraus, & Schwarzkopf, 2023), integrated assessment and treatment of co-occurring conditions are required ( Dowling et al., 2015).

Reaching a *scientific consensus on the definition and appropriate measures of at-risk gambling and problem gambling* was also considered timely. Although multiple validated tools exist to identify problem gambling ( Dowling et al., 2019), no consensus has been reached on their use and on how they should be applied in different contexts of screening, diagnosis, measurement of symptom severity or harms related to gambling ( Bowden-Jones et al., 2022).

Several research topics identified in this collaborative work would require *longitudinal research designs* to fully comprehend the temporal relationships between different gambling-related phenomena and *cross-cultural designs* to understand the role of cultural, economic, and legislative environments. Such research methods will require significant financial resources, careful planning, and collaboration from the research community.

Finally, highly relevant topics suggested by the panel that were not considered feasible and answerable need further examination, and collaborative efforts are required to find ways of exploring them. Collaborative efforts should also be supplemented by the application of open science principles in order to increase transparency, quality and replicability of research, and to ensure that results are widely available and have a meaningful impact (see Eben et al., 2023 for a discussion).

This project has limitations. While the sample of experts was balanced in terms of sex, participants from North America and Europe, and who were White, were overrepresented, although this might be representative of the characteristics of the global researcher population. There was a high percentage of psychologists and psychiatrists among the respondents, which might contribute to the predominance of the treatment perspective, as opposed to other topics such as research on policies. This also indicates that the group of researchers publishing intensively in the field is relatively homogenous in terms of academic background, and gambling research would likely benefit from having more researchers from other disciplines, such as economics, sociology, mathematics, or political science. All stakeholders participating in Phase 3 were from Australia, Europe, and North America, while other parts of the world were not represented. Also, the range of the research priority scores was relatively narrow, making it difficult to differentiate between the level of importance of the top-priority questions. Findings suggest that all top-scoring themes are highly pressing.

In conclusion, this global exercise successfully involved the gambling research community and other stakeholders in identifying research priorities. Although we used a 5-year framework to help focus on what is feasible over a short term, many of the questions that require research are complex and not quickly resolvable, thus our view is that the results of this priority setting will, in fact be relevant for a longer time frame. These results provide valuable insights for researchers, policymakers, and funding organisations. To proceed, research centres and groups should focus on these priorities and address the listed options through specific projects, and funding organisations should provide funds for their implementation. Nevertheless, in some of the more general topics, such as treatment, specific expert studies would help to reach a consensus on the most relevant sub-topics and methodological recommendations. Addressing these priorities should involve multiyear plans, collaborations, predictable funding streams and comprehensive research strategies.

## Supplementary material

## Appendix

**Table A1.** Detailed scoring and ranking of all research options

Type	Rank	Research option	Type of research option	Theme	Unweighted criteria scores								RPSs	WRPSs	AEA	Overall rank
					Answerability	Feasibility	Effectiveness	Impact on equity	Novelty	Relevance	Deliverability of intervention	Potential for burden reduction				
A Description	A1	Investigating factors related to treatment outcomes for people with gambling disorder.	description	treatment	0.898	0.830	0.879	0.806	0.699	0.901			0.836	0.848	94.9%	1
	A2	Studying the epidemiology of problem gambling in vulnerable populations (e.g., underrepresented minority groups, individuals with mental disorders or brain injuries, low-income household members, homeless individuals).	description	epidemiology	0.892	0.790	0.817	0.918	0.745	0.870			0.839	0.845	94.8%	2
	A3	Epidemiological research on gambling among adolescents and young adults.	description	epidemiology	0.930	0.878	0.823	0.777	0.609	0.824			0.807	0.819	91.9%	4
	A4	Epidemiological research on new forms of gambling.	description	epidemiology	0.860	0.833	0.785	0.706	0.872	0.850			0.818	0.817	94.4%	5
	A5	Studying the nature and harms related to newer forms of gambling and gambling-like activities (e.g., in-play betting, fantasy sports, cryptocasinos, esports betting, virtual reality gambling).	description	consequence	0.864	0.823	0.777	0.677	0.899	0.860			0.817	0.816	95.0%	6
	A6	Investigating the role of gambling-focused advertising (including sponsorship, streaming platforms, online influencers) in problem gambling among youth.	description	etiology	0.807	0.765	0.817	0.782	0.787	0.871			0.805	0.810	94.6%	7
	A7	Investigating risk and protective factors of gambling problems among adolescents and young adults.	description	etiology	0.875	0.824	0.811	0.786	0.638	0.836			0.795	0.806	92.5%	10
	A8	Investigating the treatment needs of minority populations (ethnic, cultural, linguistic, gender, sexual, immigrant, etc.).	description	treatment	0.806	0.715	0.751	0.903	0.801	0.821			0.800	0.799	95.1%	11
	A9	Assessing the impact of gambling and related harms in the case of significant others (children, partners, other family members) and investigating strategies of coping.	description	consequence	0.858	0.809	0.767	0.766	0.727	0.821			0.791	0.796	93.3%	13
	A10	Investigating the gambling behaviour and problem gambling in minority groups (ethnic, cultural, linguistic, gender, sexual, immigrant, etc.).	description	etiology	0.829	0.749	0.732	0.888	0.715	0.804			0.786	0.789	93.8%	15
	A11	Cross-cultural epidemiological studies of problem gambling (e.g., across time, different jurisdictions and countries with different economic conditions).	description	epidemiology	0.855	0.725	0.750	0.812	0.745	0.814			0.784	0.787	92.3%	17
	A12	Epidemiological research on the co-occurrence of problem gambling and non-gambling somatic, mental health and addictive disorders.	description	epidemiology/comorbidity	0.894	0.841	0.754	0.754	0.596	0.802			0.774	0.784	90.0%	19
	A13	Reaching a scientific consensus on the definition and empirically based measures of at-risk gambling and problem gambling.	description	taxonomy	0.819	0.820	0.805	0.723	0.633	0.821			0.770	0.781	88.7%	20
	A14	Studying the longitudinal relationship between gambling-like activities (e.g., loot boxes, social casino games), gambling engagement and problem gambling.	description	etiology	0.852	0.749	0.757	0.656	0.776	0.838			0.771	0.776	89.7%	22
	A15	Studying the individual and environmental factors of relapse in problem gambling.	description	treatment	0.805	0.751	0.803	0.681	0.686	0.849			0.763	0.773	90.6%	24
	A16	Investigating the roles of social media (i.e., online communities, influencers) in the development of problem gambling.	description	etiology	0.813	0.770	0.745	0.659	0.798	0.785			0.762	0.762	91.4%	33
	A17	Reaching a scientific consensus on gambling craving, its assessment and its possible inclusion in diagnostic criteria for gambling disorder.	description	taxonomy	0.817	0.807	0.798	0.581	0.701	0.805			0.752	0.760	87.3%	34
	A18	Investigating gambling-related policymaking and regulation (barriers of meaningful changes, use of empirical evidence and the involvement of multiple and specific stakeholders in the process).	description	other	0.755	0.667	0.765	0.719	0.770	0.850			0.754	0.760	90.1%	35
	A19	Establishing globally harmonized psychometric methodologies and tools for cross-cultural epidemiological research in gambling, problem gambling and gambling-related harms.	description	taxonomy	0.754	0.643	0.745	0.785	0.779	0.828			0.756	0.759	89.3%	37
	A20	Characteristics, etiologies and consequences of gambling among older adults.	description	etiology/consequence	0.848	0.782	0.715	0.772	0.675	0.728			0.753	0.756	92.3%	39
	A21	Investigating the roles of interpersonal factors (e.g., loneliness, prosocial behaviours, social support, intimate relationship quality) in problem gambling.	description	etiology	0.878	0.842	0.728	0.659	0.622	0.748			0.746	0.754	88.7%	41
	A22	Assessing the social cost and public health impact of problem gambling across countries with different cultures and regulatory policies.	description	consequence	0.740	0.656	0.725	0.776	0.775	0.831			0.751	0.753	89.3%	42
	A23	Investigating the roles of emotions and emotional regulation in the development and maintenance of problem gambling.	description	etiology	0.872	0.848	0.734	0.610	0.612	0.773			0.742	0.751	87.4%	43
	A24	Establishing the diagnostic criteria of gambling disorder in the next full revision of the DSM (i.e., “DSM-6”)	description	taxonomy	0.893	0.871	0.781	0.635	0.498	0.734			0.735	0.750	81.1%	45
	A25	Investigating the associations between online gambling and other online risky behaviours (e.g., video gaming, online sexual activities, online buying).	description	comorbidity	0.879	0.840	0.684	0.602	0.736	0.738			0.747	0.747	88.6%	47
	A26	Investigating the association between gambling type and preference and problem severity.	description	etiology	0.891	0.868	0.753	0.618	0.541	0.736			0.735	0.746	84.7%	48
	A27	Investigating the roles of game features and designs and gambling environment in the development of problem gambling.	description	etiology	0.796	0.747	0.768	0.612	0.732	0.789			0.741	0.746	88.0%	49
	A28	Investigating the roles of cognitive processes (metacognition, attention deficit, cognitive bias, decision-making, altered states of consciousness while gambling) in the development of problem gambling.	description	etiology	0.854	0.809	0.738	0.592	0.645	0.781			0.737	0.746	87.9%	50
	A29	Establishing a consensus on the definition and measurement of recovery from gambling disorder.	description	taxonomy	0.756	0.734	0.762	0.659	0.712	0.808			0.739	0.745	87.5%	51
	A30	Investigating patterns of gambling-related problems in different subgroups (e.g., age, life stage, ethnicity, gender, gambling behaviour).	description	consequence	0.844	0.782	0.676	0.791	0.608	0.721			0.737	0.741	90.1%	53
	A31	Investigating the moderating roles of psychological and cognitive variables between poor socioeconomic status and problem gambling.	description	etiology	0.786	0.763	0.674	0.774	0.674	0.755			0.738	0.740	89.6%	54
	A32	Reaching a scientific consensus regarding the clear definition of gambling, and where do we draw the borders between neighbouring, gambling	description	taxonomy	0.793	0.808	0.725	0.585	0.630	0.809			0.725	0.735	83.7%	56
	A33	Investigating the roles of sex and gender in the development of problem gambling.	description	etiology	0.884	0.866	0.655	0.771	0.492	0.689			0.726	0.734	84.0%	58
	A34	Investigating the role of problem gambling in self-harming behaviour (suicidal and non-suicidal.)	description	consequence	0.769	0.709	0.742	0.665	0.704	0.781			0.728	0.734	88.6%	59
	A35	Investigating the effects of co-occurrences between gambling disorder and other mental health and addictive disorders on the trajectory of gambling disorder.	description	comorbidity	0.803	0.701	0.710	0.638	0.677	0.794			0.721	0.728	88.8%	61
	A36	Investigating factors (biological, psychological, social) responsible for co-occurrences between gambling disorder and other mental health and addictive disorders.	description	comorbidity	0.783	0.736	0.716	0.661	0.625	0.783			0.717	0.726	86.6%	62
	A37	Studying and differentiating between harm due directly to problem gambling or to co-occurring conditions.	description	taxonomy	0.684	0.641	0.708	0.720	0.739	0.824			0.719	0.724	84.4%	64
	A38	Exploring factors associated with the continuity and discontinuity of gambling behaviour between adolescence and adulthood, and between early and middle adulthood.	description	etiology	0.781	0.655	0.678	0.642	0.764	0.798			0.720	0.723	88.1%	65
	A39	Understanding the processes and factors underlying spontaneous recovery from gambling disorder.	description	consequence	0.703	0.663	0.700	0.613	0.808	0.816			0.717	0.719	87.3%	68
	A40	Investigating stigma related to gambling.	description	other	0.824	0.783	0.643	0.726	0.621	0.694			0.715	0.718	84.8%	69
	A41	Cross-cultural research on background factors and harms related to problem gambling, including the importance of cultural and structural factors (socioeconomy, cultural norms, spirituality, cross-generational factors, etc.).	description	etiology/consequence	0.755	0.692	0.646	0.774	0.705	0.726			0.716	0.716	88.3%	70
	A42	Studying the factors (e.g., characteristics and motivations) of choosing not to gamble.	description	etiology	0.817	0.790	0.674	0.543	0.720	0.732			0.713	0.715	83.5%	71
	A43	Identifying core gambling disorder symptoms across different subgroups of people who gamble.	description	consequence	0.831	0.787	0.677	0.649	0.631	0.689			0.711	0.714	84.9%	72
	A44	Investigating the roles of stress and anxiety in problem gambling.	description	etiology	0.875	0.871	0.702	0.573	0.467	0.689			0.696	0.709	80.2%	73
	A45	Investigating the roles of childhood adversities in the development of problem gambling.	description	etiology	0.759	0.679	0.631	0.692	0.685	0.759			0.701	0.704	83.4%	74
	A46	Investigating the roles of somatic and mental health disorders in the development of problem gambling.	description	etiology	0.807	0.745	0.665	0.665	0.573	0.712			0.695	0.702	85.0%	75
	A47	Understanding the interrelated development and causal linkages of co-occurring gambling, mental health and addictive disorders.	description	comorbidity	0.715	0.607	0.705	0.631	0.684	0.782			0.687	0.694	83.9%	77
	A48	Investigating similarities and differences of gambling disorder and other behavioural and substance-related addictive disorders.	description	other	0.833	0.795	0.653	0.543	0.562	0.721			0.685	0.694	80.8%	78
	A49	Investigating characteristics, background factors and consequences of gambling-related criminal activity and violence.	description	consequence	0.714	0.650	0.643	0.656	0.689	0.706			0.676	0.677	83.0%	82
	A50	Studying the effects of gambling on the brain or neurocognitive development of adolescents.	description	consequence	0.733	0.614	0.614	0.555	0.769	0.767			0.675	0.676	78.3%	83
	A51	Comparing background factors that differentiate between disordered and non-disordered high-level involvement in gambling.	description	etiology	0.758	0.734	0.627	0.570	0.658	0.688			0.673	0.674	81.6%	84
	A52	Prevalence, types and characteristics of workplace gambling and related policies and harms in different workplace cultures.	description	epidemiology/consequence	0.773	0.718	0.630	0.605	0.712	0.628			0.678	0.674	85.4%	85
	A53	Investigating the biological and neurobiological mechanisms underlying gambling disorder.	description	etiology	0.754	0.674	0.628	0.525	0.646	0.762			0.665	0.672	78.3%	86
	A54	Defining and identifying credible, agreed-upon measures of “responsible gambling.”	description	taxonomy	0.654	0.668	0.687	0.599	0.633	0.723			0.661	0.666	77.3%	89
	A55	Investigating the roles of chasing (losses and wins) in problem gambling.	description	etiology	0.845	0.816	0.659	0.455	0.425	0.662			0.644	0.659	79.3%	90
	A56	Investigating the roles of conditioning in the development of problem gambling.	description	etiology	0.772	0.742	0.647	0.472	0.526	0.648			0.635	0.643	75.8%	93
	A57	Investigating the social representation of gambling in the general population.	description	other	0.770	0.729	0.553	0.550	0.593	0.600			0.633	0.633	73.4%	94
	A58	Investigating the roles of personality factors in problem gambling.	description	etiology	0.823	0.809	0.564	0.496	0.437	0.604			0.622	0.631	72.7%	95
	A59	Understanding the background factors and characteristics of non-problematic gambling.	description	other	0.769	0.742	0.552	0.473	0.598	0.590			0.621	0.621	71.7%	96
	A60	Studying the societal harms related to low-risk gambling.	description	consequence	0.679	0.653	0.550	0.552	0.680	0.619			0.622	0.619	73.1%	98
	A61	Investigating the genetic and epigenetic processes involved in problem gambling and its development.	description	etiology	0.674	0.590	0.535	0.504	0.720	0.679			0.617	0.615	70.9%	99
B Intervention-focused (Delivery, development and discovery type)	B1	Investigating the effectiveness of mobile/online tools that increase the accessibility of problem gambling interventions.	delivery	treatment	0.903	0.872	0.824	0.736			0.810	0.837	0.830	0.830	95.8%	3
	B2	Identifying factors that hinder treatment-seeking for problem gambling.	delivery	treatment	0.853	0.809	0.819	0.779			0.718	0.872	0.808	0.809	95.9%	8
	B3	Evaluating the effectiveness of existing psychosocial treatments for gambling disorder.	development	treatment	0.902	0.823	0.812	0.682			0.778	0.838	0.806	0.807	94.3%	9
	B4	Identifying factors behind dropping out of problem gambling treatment.	delivery	treatment	0.846	0.788	0.826	0.752			0.720	0.846	0.796	0.798	95.8%	12
	B5	Evaluating the effectiveness of existing online and mobile gambling interventions for at-risk and problem gambling.	development	prevention	0.872	0.826	0.784	0.687			0.769	0.815	0.792	0.792	93.7%	14
	B6	Evaluating the effectiveness of existing gambling problem prevention programs for adolescents and young adults.	development	prevention	0.859	0.783	0.797	0.754			0.731	0.803	0.788	0.788	95.1%	16
	B7	Formulation of evidence-based recommendations for the regulation of gambling-related advertisements.	discovery	policy	0.829	0.810	0.804	0.748			0.716	0.804	0.785	0.785	93.7%	18
	B8	Evaluating the effectiveness of existing treatments for gambling disorder co-occurring with other addictive or mental health disorders.	development	treatment	0.848	0.767	0.789	0.690			0.741	0.818	0.776	0.778	93.9%	21
	B9	Development of evidence based interventions to prevent relapse.	discovery	prevention	0.817	0.742	0.796	0.672			0.725	0.871	0.771	0.776	91.9%	23
	B10	Tailoring evidence-based treatments for subgroups of people with gambling disorder (e.g., youth, adolescents, older adults, women, minorities)	development	treatment	0.809	0.682	0.782	0.846			0.686	0.826	0.772	0.773	93.5%	25
	B11	Investigating the effectiveness of standardized treatment protocols (or deviations from them).	delivery	treatment	0.874	0.787	0.787	0.639			0.754	0.783	0.771	0.772	90.3%	26
	B12	Studying the availability of different treatment services for people with problem gambling across countries and regions.	delivery	treatment	0.873	0.797	0.749	0.796			0.673	0.770	0.776	0.771	93.3%	27
	B13	Evaluating the effectiveness of interventions for or including significant others of people who gamble or have gambling problems.	development	treatment	0.843	0.766	0.763	0.688			0.722	0.828	0.768	0.770	93.2%	28
	B14	Developing new psychosocial treatments and evidence-based guidelines for the treatment of gambling disorder.	discovery	treatment	0.833	0.765	0.777	0.680			0.735	0.808	0.766	0.768	93.7%	29
	B15	Development of easily available e-health (e.g., online, mobile, game-based, virtual reality) interventions for problem gambling.	discovery	prevention	0.835	0.755	0.745	0.705			0.773	0.781	0.766	0.766	92.6%	30
	B16	Developing evidence-based measures to facilitate treatment-seeking at an early stage of problem gambling.	delivery	treatment	0.755	0.702	0.811	0.708			0.704	0.871	0.759	0.765	94.2%	31
	B17	Identifying effective preventive tools for vulnerable populations (e.g., underrepresented minorities, women, individuals with mental disorder and brain injuries, low-income household members, homeless individuals) and improve existing preventive interventions.	development	prevention	0.769	0.689	0.776	0.876			0.649	0.818	0.763	0.762	92.0%	32
	B18	Formulation of evidence-based recommendations for the regulation of the gambling industry with the aim of minimizing individual and societal harms.	discovery	policy	0.806	0.729	0.771	0.780			0.646	0.822	0.759	0.759	89.4%	36
	B19	Evaluating the effectiveness and comparative analysis of existing gambling prevention programmes in terms of reach and effectiveness.	development	prevention	0.804	0.723	0.772	0.723			0.703	0.803	0.755	0.757	92.0%	38
	B20	Developing new forms of treatments for patients with gambling disorder that co-occur with other addictive or mental health disorders.	discovery	treatment	0.825	0.728	0.738	0.705			0.717	0.812	0.754	0.756	94.1%	40
	B21	Evaluating the effectiveness of existing behavioural-tracking-data-based detection methods of problematic gambling behaviour used by gambling operators.	development	prevention	0.810	0.710	0.790	0.686			0.702	0.786	0.747	0.751	90.3%	44
	B22	Big data and artificial intelligence (e.g., machine learning) analysis of behavioural tracking data to detect indices of risky/harmful behaviour, and use of collected data to inform prevention and harm-reduction approaches.	discovery	prevention	0.833	0.744	0.774	0.689			0.686	0.765	0.749	0.749	90.0%	46
	B23	Discovering the support needs of significant affected others and developing evidence-based targeted interventions for them.	discovery	treatment	0.814	0.737	0.739	0.704			0.664	0.804	0.744	0.744	92.9%	52
	B24	Research on the adaptation of existing evidence-based treatments for gambling disorder to account for the modern gambling landscape and new forms of gambling.	development	treatment	0.788	0.733	0.764	0.653			0.717	0.767	0.737	0.740	92.1%	55
	B25	Identifying factors that increase or hinder the use of voluntary responsible gambling tools/interventions.	delivery	prevention	0.810	0.770	0.751	0.632			0.689	0.753	0.734	0.735	89.1%	57
	B26	Formulation of evidence-based recommendations for the regulation of non-traditional gambling and gambling-like activities such as loot boxes in video games.	discovery	policy	0.800	0.734	0.735	0.704			0.650	0.768	0.732	0.731	89.4%	60
	B27	Development of youth prevention measures to reduce the risk of longer term gambling problems from new forms of gambling and gambling-like activities (such as esports and skins betting, loot boxes).	discovery	prevention	0.782	0.683	0.727	0.701			0.684	0.768	0.724	0.726	89.2%	63
	B28	Comparing the effectiveness of different treatment approaches of problem gambling at all levels of severity.	development	treatment	0.775	0.644	0.763	0.629			0.674	0.799	0.714	0.720	85.8%	66
	B29	Critical analysis of gambling policies of different jurisdictions (including public health approach and responsible gambling policies), their changes over time, their effectiveness, connection to prevalence of problem gambling and gambling-related harm.	development	policy	0.753	0.696	0.740	0.734			0.635	0.756	0.719	0.719	87.9%	67
	B30	Investigating key elements of effective communication with people who gamble in the prevention of problem gambling.	development	prevention	0.714	0.693	0.725	0.655			0.664	0.711	0.694	0.695	88.1%	76
	B31	Evaluating the effectiveness of existing pharmaceutical interventions for gambling disorder.	development	treatment	0.837	0.753	0.685	0.520			0.691	0.669	0.693	0.692	80.5%	79
	B32	Understanding background factors of problem gambling among military personnel and veterans and tailoring preventive measures.	development	prevention	0.820	0.727	0.640	0.703			0.625	0.671	0.698	0.691	83.3%	80
	B33	Evaluating the effectiveness and comparative analysis of responsible gambling strategies and tools used by operators and possible ways to improve these.	development	prevention	0.741	0.656	0.710	0.622			0.634	0.750	0.686	0.689	82.9%	81
	B34	Developing methodologies to identify people with at-risk gambling across countries and operators using a standardized approach.	discovery	prevention	0.716	0.602	0.678	0.698			0.599	0.714	0.668	0.669	82.3%	87
	B35	Development of joint internet risk prevention programmes including multiple behaviours such as online gambling, video gaming.	discovery	prevention	0.736	0.657	0.645	0.612			0.654	0.695	0.667	0.667	82.9%	88
	B36	Constructing a system/classification scale for assessing new forms of gambling for potential burden of harm before they are released to market.	discovery	prevention	0.660	0.576	0.695	0.638			0.598	0.751	0.653	0.659	77.3%	91
	B37	Evaluating the effectiveness of existing neuromodulation (neurofeedback, transcranial direct current stimulation (tDCS) and transcranial magnetic stimulation (TMS)) interventions for problem gambling.	development	treatment	0.815	0.713	0.650	0.478			0.600	0.624	0.647	0.645	76.8%	92
	B38	Developing new pharmacological interventions for gambling disorder.	discovery	treatment	0.748	0.614	0.601	0.488			0.600	0.658	0.618	0.620	74.1%	97
	B39	Finding an effective method to calculate the maximum amount of money a specific player can lose in a given time period, to improve responsible gambling tools and their implementation.	discovery	policy	0.612	0.593	0.586	0.536			0.566	0.636	0.588	0.590	66.2%	100
	B40	Studying the applicability of artificial intelligence to support diagnosis.	discovery	treatment	0.677	0.594	0.578	0.509			0.574	0.586	0.586	0.586	67.9%	101
	B41	Exploring new treatment approaches for gambling disorder, such as noninvasive neuromodulation (repetitive transcranial magnetic stimulation, transcranial electrical stimulation, etc.) and deep brain stimulation.	discovery	treatment	0.746	0.602	0.576	0.458			0.513	0.614	0.585	0.584	68.7%	102

RPS = Research Priority Score, WRPS = Weighted Research Priority Score, AEA = average expert agreement.

## References

[B1] Abbott, M. , Binde, P. , Clark, L. , Hodgins, D. , Johnson, M. , Manitowabi, D. , … Williams, R. ( 2018). *Conceptual framework of harmful gambling* ( 3rd ed.). Gambling Research Exchange Ontario. 10.33684/CFHG3.en https://doi.org/10.33684/CFHG3.en PMC8939413 32554839

[B2] American Psychiatric Association ( 2022). *Diagnostic and statistical Manual of mental disorders (DSM-5-TR)* . American Psychiatric Association Publishing. 10.1176/appi.books.9780890425787 https://doi.org/10.1176/appi.books.9780890425787

[B3] Armitage, R. ( 2021). Gambling among adolescents: An emerging public health problem. *The Lancet Public Health* , 6( 3), e143. 10.1016/S2468-2667(21)00026-8 https://doi.org/10.1016/S2468-2667(21)00026-8 33640074

[B4] ATLAS.ti Scientific Software Development GmbH ( 2023). *ATLAS.ti Web* . [Computer software]. Retrieved from https://atlasti.com.

[B5] Bijker, R. , Booth, N. , Merkouris, S. S. , Dowling, N. A. , & Rodda, S. N. ( 2022). Global prevalence of help‐seeking for problem gambling: A systematic review and meta‐analysis. *Addiction* , 117( 12), 2972– 2985. 10.1111/add.15952 https://doi.org/10.1111/add.15952 35830876 PMC9796401

[B6] Bowden-Jones, H. , Hook, R. W. , Grant, J. E. , Ioannidis, K. , Corazza, O. , Fineberg, N. A. , … Chamberlain, S. R. ( 2022). Gambling disorder in the UK: Key research priorities and the urgent need for independent research funding. *The Lancet Psychiatry* , 9( 4), 321– 329. 10.1016/S2215-0366(21)00356-4 https://doi.org/10.1016/S2215-0366(21)00356-4 35180386 PMC7612512

[bib44] Brand, M. , Antons, S. , Bőthe, B. , Demetrovics, Z. , Fineberg, N. A. , Jimenez-Murcia, S. , ... Potenza, M. N. ( 2025). Current Advances in Behavioral Addictions: From Fundamental Research to Clinical Practice. *American Journal of Psychiatry* , 182( 2), 155– 163. 10.1176/appi.ajp.20240092 https://doi.org/10.1176/appi.ajp.20240092 39659159

[B7] Browne, M. , Langham, E. , Rawat, V. , Greer, N. , Li, E. , Rose, J. , … Best, T. ( 2016, April). Assessing gambling-related harm in Victoria: A public health perspective. *Victorian Responsible Gambling Foundation, Melbourne* Retrieved from https://apo.org.au/sites/default/files/resource-files/2016-04/apo-nid62714.pdf.

[B8] Browne, M. , & Rockloff, M. J. ( 2018). Prevalence of gambling-related harm provides evidence for the prevention paradox. *Journal of Behavioral Addictions* , 7( 2), 410– 422. 10.1556/2006.7.2018.41 https://doi.org/10.1556/2006.7.2018.41 29788761 PMC6174604

[B9] Calado, F. , Alexandre, J. , & Griffiths, M. D. ( 2017). Prevalence of adolescent problem gambling: A systematic review of recent research. *Journal of Gambling Studies* , 33( 2), 397– 424. 10.1007/s10899-016-9627-5 https://doi.org/10.1007/s10899-016-9627-5 27372832 PMC5445143

[B10] Collins, P. Y. , Patel, V. , Joestl, S. S. , March, D. , Insel, T. R. , Daar, A. S. , … Walport, M. ( 2011). Grand challenges in global mental health. *Nature* , 475( 7354), 27– 30. 10.1038/475027a https://doi.org/10.1038/475027a 21734685 PMC3173804

[B11] Dowling, N. A. , Cowlishaw, S. , Jackson, A. C. , Merkouris, S. S. , Francis, K. L. , & Christensen, D. R. ( 2015). Prevalence of psychiatric co-morbidity in treatment-seeking problem gamblers: A systematic review and meta-analysis. *Australian & New Zealand Journal of Psychiatry* , 49( 6), 519– 539. 10.1177/0004867415575774 https://doi.org/10.1177/0004867415575774 25735959 PMC4438101

[B12] Dowling, N. A. , Merkouris, S. S. , Dias, S. , Rodda, S. N. , Manning, V. , Youssef, G. J. , … Volberg, R. A. ( 2019). The diagnostic accuracy of brief screening instruments for problem gambling: A systematic review and meta-analysis. *Clinical Psychology Review* , 74, 101784. 10.1016/j.cpr.2019.101784 https://doi.org/10.1016/j.cpr.2019.101784 31759246

[B13] Eben, C. , Bőthe, B. , Brevers, D. , Clark, L. , Grubbs, J. B. , Heirene, R. , … Billieux, J. ( 2023). The landscape of open science in behavioral addiction research: Current practices and future directions. *Journal of Behavioral Addictions* , 12( 4), 862– 870. 10.1556/2006.2023.00052 https://doi.org/10.1556/2006.2023.00052 38141055 PMC10786235

[B14] Emond, A. M. , & Griffiths, M. D. ( 2020). Gambling in children and adolescents. *British Medical Bulletin* , 136( 1), 21– 29. 10.1093/bmb/ldaa027 https://doi.org/10.1093/bmb/ldaa027 32932525

[B15] Fineberg, N. A. , Demetrovics, Z. , Stein, D. J. , Ioannidis, K. , Potenza, M. N. , Grünblatt, E. , … Chamberlain, S. R. ( 2018). Manifesto for a European research network into problematic usage of the internet. *European Neuropsychopharmacology: The Journal of the European College of Neuropsychopharmacology* , 28( 11), 1232– 1246. 10.1016/j.euroneuro.2018.08.004 https://doi.org/10.1016/j.euroneuro.2018.08.004 30509450 PMC6276981

[B16] Gartner, C. , Bickl, A. , Härtl, S. , Loy, J. K. , & Häffner, L. ( 2022). Differences in problem and pathological gambling: A narrative review considering sex and gender. *Journal of Behavioral Addictions* , 11( 2), 267– 289. 10.1556/2006.2022.00019 https://doi.org/10.1556/2006.2022.00019 35499928 PMC9295224

[bib45] Gjoneska, B. , Bőthe, B. , Potenza, M. N. , Szabo, A. , & Demetrovics, Z. ( 2025). Epidemiology of behavioral addictions. In I. Franken , R. Wiers , & K. Witkiewitz (Eds.), *The Sage Handbook of Addiction Psychology* (pp. 47– 62). Sage Publications Ltd. 10.4135/9781529673913.n4 https://doi.org/10.4135/9781529673913.n4

[B17] Grall-Bronnec, M. , Guillou-Landreat, M. , Caillon, J. , Dubertret, C. , Romo, L. , Codina, I. , Chereau-Boudet, I. , Lancon, C. , Auriacombe, M. , Hardouin, J.-B. , & Challet-Bouju, G. ( 2021). Five-year follow-up on a sample of gamblers: Predictive factors of relapse. *Journal of Behavioral Addictions* , 10( 1), 42– 54. 10.1556/2006.2021.00009 https://doi.org/10.1556/2006.2021.00009 33793415 PMC8969856

[B18] Grant, J. E. , & Chamberlain, S. R. ( 2023). Gambling disorder in minority ethnic groups. *Addictive Behaviors* , 136, 107475. 10.1016/j.addbeh.2022.107475 https://doi.org/10.1016/j.addbeh.2022.107475 36081247 PMC7613642

[B19] Hartmann, M. , & Blaszczynski, A. ( 2018). The longitudinal relationships between psychiatric disorders and gambling disorders. *International Journal of Mental Health and Addiction* , 16( 1), 16– 44. 10.1007/s11469-016-9705-z https://doi.org/10.1007/s11469-016-9705-z

[B20] Hing, N. , Russell, A. M. T. , Browne, M. , Rockloff, M. , Tulloch, C. , Rawat, V. , … Woo, L. ( 2022). Gambling-related harms to concerned significant others: A national Australian prevalence study. *Journal of Behavioral Addictions* , 11( 2), 361– 372. 10.1556/2006.2022.00045 https://doi.org/10.1556/2006.2022.00045 35895474 PMC9295213

[B21] Király, O. , Zhang, J. , Demetrovics, Z. , & Browne, D. T. ( 2021). Gambling features and monetization in video games creates challenges for young people, families, and clinicians. *Journal of the American Academy of Child and Adolescent Psychiatry* , S089085672102044X. 10.1016/j.jaac.2021.12.003 https://doi.org/10.1016/j.jaac.2021.12.003 34921907

[B22] Langham, E. , Thorne, H. , Browne, M. , Donaldson, P. , Rose, J. , & Rockloff, M. ( 2015). Understanding gambling related harm: A proposed definition, conceptual framework, and taxonomy of harms. *BMC Public Health* , 16( 1), 80. 10.1186/s12889-016-2747-0 https://doi.org/10.1186/s12889-016-2747-0 PMC4728872 26818137

[B23] Lee, B. N. , & Grubbs, J. B. ( 2023). Problem gambling within sexual and gender minorities: A systematic review. *Addictive Behaviors* , 144, 107742. 10.1016/j.addbeh.2023.107742 https://doi.org/10.1016/j.addbeh.2023.107742 37121088

[B24] Moreira, D. , Azeredo, A. , & Dias, P. ( 2023). Risk factors for gambling disorder: A systematic review. *Journal of Gambling Studies* , 39( 2), 483– 511. 10.1007/s10899-023-10195-1 https://doi.org/10.1007/s10899-023-10195-1 36884150 PMC9994414

[B25] Okuda, M. , Liu, W. , Cisewski, J. A. , Segura, L. , Storr, C. L. , & Martins, S. S. ( 2016). Gambling disorder and minority populations: Prevalence and risk factors. *Current Addiction Reports* , 3( 3), 280– 292. 10.1007/s40429-016-0108-9 https://doi.org/10.1007/s40429-016-0108-9 28824833 PMC5560497

[B26] Petry, N. M. , Stinson, F. S. , & Grant, B. F. ( 2005). Comorbidity of DSM-IV pathological gambling and other psychiatric disorders: Results from the national epidemiologic survey on alcohol and related conditions. *The Journal of Clinical Psychiatry* , 66( 05), 564– 574. 10.4088/JCP.v66n0504 https://doi.org/10.4088/JCP.v66n0504 15889941

[B27] Pfund, R. A. , Peter, S. C. , McAfee, N. W. , Ginley, M. K. , Whelan, J. P. , & Meyers, A. W. ( 2021). Dropout from face-to-face, multi-session psychological treatments for problem and disordered gambling: A systematic review and meta-analysis. *Psychology of Addictive Behaviors* , 35( 8), 901– 913. 10.1037/adb0000710 https://doi.org/10.1037/adb0000710 34881915 PMC8666798

[B28] Rudan, I. , Chopra, M. , Kapiriri, L. , Gibson, J. , Lansang, M. A. , Carneiro, I. , … Black, R. E. ( 2008). Setting priorities in global child health research investments: Universal challenges and conceptual framework. *Croatian Medical Journal* , 49( 3), 307– 317. 10.3325/cmj.2008.3.307 https://doi.org/10.3325/cmj.2008.3.307 18581609 PMC2443616

[B29] Rudan, I. , Gibson, J. L. , Ameratunga, S. , El Arifeen, S. , Bhutta, Z. A. , Black, M. , … Webster, J. ( 2008). Setting priorities in global child health research investments: Guidelines for implementation of the CHNRI method. *Croatian Medical Journal* , 49( 6), 720– 733. 10.3325/cmj.2008.49.720 https://doi.org/10.3325/cmj.2008.49.720 19090596 PMC2621022

[B30] Rudan, I. , Yoshida, S. , Chan, K. Y. , Sridhar, D. , Wazny, K. , Nair, H. , … Cousens, S. ( 2017). Setting health research priorities using the CHNRI method: VII. A review of the first 50 applications of the CHNRI method. *Journal of Global Health* , 7( 1), 011004. 10.7189/jogh.07.011004 https://doi.org/10.7189/jogh.07.011004 28685049 PMC5481891

[B31] Swanton, T. B. , Blaszczynski, A. , Forlini, C. , Starcevic, V. , & Gainsbury, S. M. ( 2021). Problematic risk-taking involving emerging technologies: A stakeholder framework to minimize harms. *Journal of Behavioral Addictions* , 9( 4), 869– 875. 10.1556/2006.8.2019.52 https://doi.org/10.1556/2006.8.2019.52 31537086 PMC8969716

[B32] Tomlinson, M. ( 2009). Setting priorities for global mental health research. *Bulletin of the World Health Organization* , 87( 6), 438– 446. 10.2471/BLT.08.054353 https://doi.org/10.2471/BLT.08.054353 19565122 PMC2686213

[B33] Torrance, J. , John, B. , Greville, J. , O’Hanrahan, M. , Davies, N. , & Roderique-Davies, G. ( 2021). Emergent gambling advertising; a rapid review of marketing content, delivery and structural features. *BMC Public Health* , 21( 1), 718. 10.1186/s12889-021-10805-w https://doi.org/10.1186/s12889-021-10805-w 33849493 PMC8043759

[B34] Tran, L. T. , Wardle, H. , Colledge-Frisby, S. , Taylor, S. , Lynch, M. , Rehm, J. , … Degenhardt, L. ( 2024). The prevalence of gambling and problematic gambling: A systematic review and meta-analysis. *The Lancet Public Health* , S2468266724001269. 10.1016/S2468-2667(24)00126-9 https://doi.org/10.1016/S2468-2667(24)00126-9 39025095

[B35] Tulloch, C. , Browne, M. , Hing, N. , Rockloff, M. , & Hilbrecht, M. ( 2023). How gambling harms others: The influence of relationship-type and closeness on harm, health, and wellbeing. *Journal of Behavioral Addictions* , 12( 3), 697– 710. 10.1556/2006.2023.00036 https://doi.org/10.1556/2006.2023.00036 37450370 PMC10562824

[B36] Ukhova, D. , Marionneau, V. , Nikkinen, J. , & Wardle, H. ( 2024). Public health approaches to gambling: A global review of legislative trends. *The Lancet Public Health* , 9( 1), e57– e67. 10.1016/S2468-2667(23)00221-9 https://doi.org/10.1016/S2468-2667(23)00221-9 37944544 PMC10927617

[B37] Wardle, H. , Degenhardt, L. , Ceschia, A. , & Saxena, S. ( 2021). The lancet public health commission on gambling. *The Lancet Public Health* , 6( 1), e2– e3. 10.1016/S2468-2667(20)30289-9 https://doi.org/10.1016/S2468-2667(20)30289-9 33417844

[B38] Wardle, H. , Degenhardt, L. , Marionneau, V. , Reith, G. , Livingstone, C. , Sparrow, M. , … Saxena, S. ( 2024). The lancet public health commission on gambling. *The Lancet Public Health* , 9( 11), e950– e994. 10.1016/S2468-2667(24)00167-1 https://doi.org/10.1016/S2468-2667(24)00167-1

[B39] West, R. , Hao, W. , Lam, T. H. , Lau, J. , Li, J. , Li, J. , … Zhao, M. ( 2019). Addiction in China: Towards a research agenda for the next 5 years. *Addiction (Abingdon, England)* , 114( 11), 1911– 1914. 10.1111/add.14650 https://doi.org/10.1111/add.14650 31081567

[B40] World Health Organization ( 2019). *International Classification of Diseases* ( 11th ed.). Retrieved from https://icd.who.int/.

[B41] Wullinger, P. M. , Bickl, A. M. , Loy, J. K. , Kraus, L. , & Schwarzkopf, L. ( 2023). Longitudinal associations between psychiatric comorbidity and the severity of gambling disorder: Results from a 36-month follow-up study of clients in Bavarian outpatient addiction care. *Journal of Behavioral Addictions* , 12( 2), 535– 546. 10.1556/2006.2023.00026 https://doi.org/10.1556/2006.2023.00026 37307216 PMC10316174

[B42] Young People and Gambling ( 2023). *Gambling Commission* . Retrieved from https://www.gamblingcommission.gov.uk/report/young-people-and-gambling-2023.

[B43] Zendle, D. , & Cairns, P. ( 2018). Video game loot boxes are linked to problem gambling: Results of a large-scale survey. *Plos One* , 13( 11), e0206767. 10.1371/journal.pone.0206767 https://doi.org/10.1371/journal.pone.0206767 30462669 PMC6248934

