# Supplementary material for: Research priorities in gambling: Findings of a large-scale expert study
Source: J Behav Addict. 2025 Sep 30;14(3):1222–49. doi: 10.1556/2006.2025.00072 (PMC12486284; doi:10.1556/2006.2025.00072)
Supplement: Supplementary file 1 [file jba-14-1222-s001.pdf]

Czakó, A. et al.: Research priorities in gambling: Findings of a large-scale expert study.
https://doi.org/10.1556/2006.2025.00072
Supplementary material

Members of the Gambling Research Priority Setting Consortium

First name	Middle name	Surname	Primary affiliation	Secondary affiliation	Tertiary affiliation	Quaternary affiliation	ORCID ID	Conflict of interest statement	Funding statement
Brett		Abarbanel	International Gaming Institute, University of Nevada, Las Vegas, USA	William F. Harrah College of Hospitality, University of Nevada, Las Vegas, USA	Science, Brain, and Mind Centre, University of Sydney, Camperdown, NSW, Australia		0000-0002-4279-8466	N/A	During the past five years, BA has received funding for research and/or consulting services from the Sports Betting Alliance, GLG Consulting, MGM Resorts International, Eilers & Krejcik Gaming, ProPress Germany, Scientific Affairs, McGill University, University of North Carolina School of Social Work, Marina Bay Sands, Aristocrat Gaming, Pixel United, Life Works, Jones Ward, Navigation Media, Aristocrat Leisure Limited, Sit Investment Associates, and Deutsche Stiftung Gluecksspielforschung. Dr. Abarbanel has received reimbursement for travel from Scientific Affairs, Deutsche Stiftung Gluecksspielforschung, British Columbia Lottery Corporation, International Association of Gaming Advisors, Sit Investment Associates, Columbia University, University of Liverpool, and University of Salford. None of these entities played a role in the design, analysis, or interpretation of this study, and impose no constraints on publishing.
Lucy		Albertella	BrainPark, Turner Institute for Brain and Mental Health, Monash University, Clayton Australia				0000-0001-5232-6414	No conflict of interest	N/A
Jean-Marc		Alexandre	Sleep Addiction and Neuropsychiatry (SANPSY) Laboratory, CNRS UMR 6033 University of Bordeaux, Bordeaux, France	Pôle Interétablissement d'Addictologie, CH Charles Perrens and CHU Bordeaux, Bordeaux, France			0000-0001-9260-903X	No conflict of interest	N/A

Youssef		Allami	School of Psychology, Laval University, Quebec, Quebec				0000-0001-7609-2405	Youssef Allami has received consulting fees from the social responsibility division of a provincial gambling operator (Crown Corporation) in Canada.	N/A
Matteo		Aloi	Department of Clinical and Experimental Medicine, University of Messina, Messina, Italy.				0000-0001-5585-6488	No conflict of interest	N/A
Laura		Angioletti	International research center for Cognitive Applied Neuroscience (IrcCAN), Università Cattolica del Sacro Cuore, Milan, Italy	Research Unit in Affective and Social Neuroscience Department of Psychology, Università Cattolica del Sacro Cuore, Milan, Italy			0000-0002-3027-2272	No conflict of interest	Laura Angioletti receives funding from Fondazione Cariplo, grant n° 2023-1612.
Wen	Li	Anthony	Center for Gambling Studies, School of Social Work, Rutgers University, New Brunswick, NJ, USA				0000-0003-1631-0161	No conflict of interest	N/A
Núria		Aragay	Behavioural Addictions Unit, Mental Health Care Area, Consorci Sanitari de Terrassa, Spain	Facultad de Medicina y ciencias de la Salud, Universidad Internacional de Catalunya, Barcelona, España.			0000-0002-0082-0303	No conflict of interest	N/A
Cecilia		Åslund	Centre for Clinical Research, Region Västmanland, Uppsala University, Uppsala, Sweden	Department of Public Health and Caring Sciences, Uppsala University, Uppsala, Sweden			0000-0002-3589-6113	No conflict of interest	Cecilia Åslund has received funding from Svenska Spel Research Council (dnr FO2015-0010, FO2019-0004, 2022-009).
Marc		Auriacombe	Sleep, Addiction and Neuropsychiaty (SANPSY) Laboratory, CNRS UMR 6033, University of Bordeaux, Bordeaux, France	Pôle Interétablissement d'Addictologie, CH Charles Perrens and CHU Bordeaux, Bordeaux, France			0000-0002-8938-8683	No conflict of interest	N/A
Larry	Okechukwu	Awo	School of General Studies, Federal Polytechnic of Oil and Gas, Bonny, Nigeria	Cognitive and Affective Cognition Laboratory, Institute of Psychology, University of Lausanne,Lausanne, Switzerland			0000-0003-2782-1151	No conflict of interest	N/A
Stéphanie		Baggio	Institute of Psychology, University of Lausanne, Lausanne, Switzerland	Institute of Primary Health Care (BIHAM), University of Bern, Bern, Switzerland			0000-0002-5347-5937	No conflict of interest	N/A
Michael	F	Baigent	Statewide Gambling Therapy Service, Southern Adelaide Local Health Network, Mental Health Service, Flinders Medical Centre, Flinders Drive, Bedford Park, South Australia, Australia	Discipline of Psychiatry, College of Medicine and Public Health, Flinders University, Adelaide, Australia			0000-0001-8815-3009	No conflict of interest	N/A

Iris	M.	Balodis	Peter Boris Centre for Addictions Research, Hamilton, ON, Canada	Department of Psychiatry and Behavioural Neurosciences, Hamilton, ON, Canada	Center for Clinical Neurosciences, Hamilton, ON, Canada	St. Joseph's Healthcare Hamilton, Hamilton, ON, Canada	0000-0001-8875-9668	No conflict of interest	Iris M. Balodis received funding from the International Centre for Responsible Gaming and the Gambling Research Exchange of Ontario
Servane		Barrault	Laboratory QualiPsy, EE1901, Psychology Department, University of Tours, ours, France	CSAPA 37, CHRU of Tours, ours, France	University of Paris, Laboratory of Psychopathology and Health Processes UR4057, Boulogne-Billancourt, France		0000-0002-4743-3087	No conflict of interest	N/A
Rafał	Piotr	Bartczuk	Institute of Psychology, John Paul II Catholic University of Lublin, Lublin, Poland	Scientific Research and International Cooperation Division, Children's Memorial Health Institute, Warsaw, Poland			0000-0002-0433-7327	No conflict of interest	N/A
Malcolm		Battersby	Discipline of Psychiatry, College of Medicine and Public Health, Flinders University, Adelaide, 5042, South Australia, Australia	Statewide Gambling Therapy Service, Southern Adelaide Local Health Network, Mental Health Service, Flinders Medical Centre, Flinders Drive, Bedford Park, South Australia, Australia			0000-0001-7306-5591	Malcolm Battersby is a co-developer of the Flinders CBT program for gambling disorders	Malcolm Battersby is a clinician researcher with the Statewide Gambling Therapy Service funded by the South Australian Department of Human Service
Maria	E.	Bellringer	Gambling and Addictions Research Centre, Faculty of Health and Environmental Sciences, Auckland University of Technology, Auckland, New Zealand				0000-0002-4675-9704	No conflict of interest	N/A
Anne H		Berman	Department of Psychology, Uppsala University, Uppsala, Sweden	Centre for Psychiatry Research, Department of Clinical Neuroscience, Karolinska Institutet, & Stockholm Health Care Services, Region Stockholm, Sweden			0000-0002-7709-0230	Anne H Berman has no current conflicts of interest. She was previously (2013-2022) a member of the independent research council funded by the state-owned gambling operator of Sweden, Svenska Spel AB, assessing applications for gambling-related research funding. She has also previously been a recipient of research funding from the research council of the state-owned alcohol monopoly in Sweden, Systembolaget AB.	N/A
Mónica		Bernaldo-De-Quirós	Department of Clinical Psychology. Complutense University of Madrid, Madrid, Spain				0000-0002-2200-8375	No conflict of interest	N/A
Roshan		Bhad	Additional Professor of Psychiatry, National Drug Dependence Treatment	Board of Director, International Society of Addiction Medicine (ISAM)			0000-0003-4824-3346	No conflict of interest	N/A

			Centre (NDDTC), AIIMS, New Delhi, India						
Alexander		Blaszczynski	School of Psychology, University of Sydney, Camperdown, NSW Australia	Brain and Mind Center, University of Sydney, Camperdown, NSW Australia			0000-0003-1476-0791	No conflict of interest	N/A
Marilisa		Boffo	Department of Psychology, Education and Child Studies, Erasmus School of Social and Behavioral Sciences, Erasmus University Rotterdam, Rotterdam, NL.				0000-0003-4730-7838	Marilisa Boffo's research is supported by internal university funding and a grant by the International Center for Responsible Gaming, in combination with financial and in-kind contribution by the gambling industry (Playtech, Holland Casino Online). Collaboration with the gambling industry is formalised in a collaboration agreement, protecting scientific integrity, independence and freedom - and ensure compliance with the Netherlands Code of Conduct for Research Integrity. The gambling industry has no influence on any scientific activity, dissemination and publication of results deriving from this research.	Marilisa Boffo has received funding from the International Center for Responsible Gaming, University of Amsterdam Valorisation fund, and the gambling industry. These organisations had no influence on the answers given for this project.
Céline		Bonnaire	Université Paris Cité, Laboratoire de Psychopathologie et Processus de Santé, F-92100 Boulogne-Billancourt, France	Centre Pierre Nicole, « Consultation Jeunes Consommateurs », Croix-Rouge Française, Paris, France			0000-0001-7162-2657	No conflict of interest	N/A
Marco		Bortolato	Department of Pharmacodynamics at the University of Florida, USA				0000-0002-4498-9637	No conflict of interest	Marco Bortolato is or has been the principal investigator of several research projects funded by NIMH, NINDS, NIDA, and NIAAA.
Stephane		Bouchard	Departement de psychoeducation et psychologie, Universite du Quebec en Outaouais, Gatineau, Qc, Canada	Research Center of the Centre Intégré de Santé et de Services Sociaux de l'Outaouais, Gatineau, Qc, Canada.	École de psychologie, Université D'Ottawa, Ottawa, Ont, Canada	École de Psychologie, Université Laval, Québec, Qc, Canada.	0000-0002-5995-340X	Stephane Bouchard is president and owner of In Virtuo, a company that distributes virtual environments for the treatment of mental disorders. Conflicts of interest are managed under UQO's conflicts of interest policy.	Stephane Bouchard received funding from the Canada research Chairs program.
Amy	E.	Bouchard	Department of Psychiatry and Neurosciences, Faculty of Medicine, Université Laval, Quebec City, Quebec, Canada	CERVO Brain Research Centre, Centre Intégré Universitaire de Santé et de Services Sociaux de la Capitale-Nationale, Quebec City, Quebec, Canada			0000-0001-7423-895X	No conflict of interest	N/A

Matthias		Brand	General Psychology: Cognition and Center for Behavioral Addiction Research (CeBAR), University of Duisburg-Essen, Duisburg, Germany	Erwin L. Hahn Institute for Magnetic Resonance Imaging, Essen, Germany			0000-0002-4831-9542	Dr. Brand is spokesperson of the Research Unit FOR2974 “Affective and cognitive mechanisms of internet-use disorders” funded by the Deutsche Forschungsgemeinschaft (DFG, German Research Foundation). He also receives funding from the EU and the German Federal Ministry of Education and Research. He has performed grant reviews for research-funding agencies; has edited journals and journal sections; has given academic lectures in clinical or scientific venues; and has generated book chapters for publishers of mental health texts.	Matthias Brand receives funding from the Deutsche Forschungsgemeinschaft (DFG, German Research Foundation) for the Research Unit ACSID, FOR2974, Pr.-Nr. 411232260.
Helen		Breen	Adjunct, Faculty of Business, Law and Arts, Southern Cross University, Lismore, Australia.				0000-0002-1350-6129	No conflict of interest	N/A
Tim	Bastian	Brosowski	Institute of Public Health and Nursing Research, University of Bremen, Germany				0000-0003-1703-1797	No conflict of interest	N/A
Matthew		Browne	CQUniversity School of Medical, Health & Applied Sciences, Bundaberg, Australia				0000-0002-2668-6229	No conflict of interest	Matthew Browne has received research funding from Australian and New Zealand governmental and statutory authorities. He has not received funding from industry sources.
Tony	W.	Buchanan	Department of Psychology, Saint Louis University, Saint Louis, MO USA				0000-0002-9166-8457	No conflict of interest	N/A
Ursula	Gisela	Buchner	German University of Health and Sports, Berlin, Germany				0000-0002-1647-9867	No conflict of interest	N/A
Gerhard		Bühringer	Work group Addictive Behaviors, Risk Analysis and Risk Management, Faculty of Psychology, Technische Universitaet Dresden, Germany				0000-0002-5568-1435	Gerhard Bühringer received unrestricted grants for gambling research activities from public and commercial gambling providers and regulatory agencies in Germany . He is a member of the ‘Düsseldorfer Kreis’ (a group of key stakeholders from public and private gambling providers, research, and the support system) and is also partly funded by an unrestricted research grant from the Federal Ministry of Economic Affairs and Climate Action as part of the evaluation of gambling hall regulations.	N/A

Alessandra		Buja	Department of Cardiologic, Vascular and Thoracic Sciences, and Public Health, University of Padua, Padua, Italy.				0000-0003-2216-3807	No conflict of interest	N/A
Natale		Canale	Department of Developmental and Social Psychology; University of Padova, Padova, Italy				0000-0002-6032-0490	No conflict of interest	N/A
Rene		Carbonneau	Department of Pediatrics, University of Montreal, Montreal, Quebec, Canada	Sainte-Justine Hospital Research Center, Montreal, Quebec, Canada	Research Unit on Children's Psychosocial Maladjustment, Montréal, Québec, Canada		0000-0003-2971-6339	No conflict of interest	N/A
Sari		Castrén	Finnish Institute for Health and Welfare, P.O. Box 30, 00271 Helsinki, Finland	Social Sciences Department of Psychology and Speech-Language Pathology Turku, University of Turku, 20014 Turku, Finland	Department of Medicine, University of Helsinki, 00014 Helsinki, Finland		0000-0003-0492-9610	Sari Castrén works a part time private practitioner clinical psychologist at Addiktum Clinic Helsinki, Finland, treating mainly individuals with addiction problems, and at Mehiläinen Medical Center, Forum Helsinki, where she offers treatments to various psychological issues. She has received fees from Helsinki University, Tampere City, Vocational school Stadi, Lundbeck, the Finnish Association of Addiction Medicine, the Finnish Association on Intellectual and Developmental Disabilities (FAIDD), and Mehiläinen for her lectures on behavioural addictions and for training professionals, and writer's fees from the Finnish Medical Society Duodecim, Finnish Medical Journal and Myllyhoitoyhdistys ry. She received fees from Svenska Spel (Sweden) for evaluating grant proposals, and Tampere University for preliminary examination of PhD work, acting as an opponent for PhD thesis (Lund University, Sweden and Bergen University, Norway). She declares no conflict of interest in relation to this manuscript.	The daily work of Sari Castrén is funded by the Ministry of Social Affairs and Health, Finland, within the objectives of Section 52 Appropriation of the Lotteries Act.
Maris		Catania	Kindred Group, Sliema, Malta	SG:Certified, London, UK			0000-0001-5103-8701	Maris Catania works as a consultant with different organisations, including Kindred Group Plc, SG:Certified and works on multiple projects with online gambling operators and universities.	N/A

Gaëlle		Challet-Bouju	Nantes Université, CHU Nantes, UIC Psychiatrie et Santé Mentale, Nantes, France	Nantes Université, Univ Tours, CHU Nantes, INSERM, MethodS in Patient-centered outcomes and HEalth ResEarch, SPHERE, Nantes, France			0000-0002-2238-8005	Gaëlle Challet-Bouju has declared that the Endowment Fund of the University Hospital of Nantes received funding from the gambling industry (FDJ and PMU) as part of the implementation of the obligation to finance scientific studies on gambling and related addictive disorders (Law n° 2010-476 of May 12th modified, art. 3). This funding does not concern the present study, has never had any influence on the present work and scientific independence, objectivity and impartiality is guaranteed	N/A
Heather	A.	Chapman	Department of Veterans Affairs, Cleveland VA Medical, 10701 e blvd cleveland ohio 44106, USA	Case Western Reserve University, Department of Psychiatry, cleveland, oh, USA			0000-0001-5819-2824	No conflict of interest	Heather A. Chapman receives funding from Kindbridge and received funding from NCRG in the past.
Emeline		Chauchard	Nantes Université, Univ Angers, Laboratoire de psychologie des Pays de la Loire, Nantes, France	UMR 7295 Centre de Recherches sur la Cognition et l'Apprentissage, CNRS, Université de Tours, Université de Poitiers, France			0000-0002-2907-6047	No conflict of interest	N/A
Juliet Honglei		Chen	Department of Psychology, Zhejiang Sci-Tech University, Hangzhou, China				0000-0002-6564-5390	No conflict of interest	N/A
Samwook		Choi	Dept. of Psychiatry, True Mind Mental Health Clinic , Gangnam-gu, Seoul, Korea	Adjunct Professor, Dept. of Psychiatry, University of Ulsan College of Medicine, Asan Medical Center, Songpa-gu, Seoul, South Korea			0000-0002-9544-7766	No conflict of interest	N/A
Jung-Seok		Choi	Department of Psychiatry, Samsung Medical Center, Sungkyunkwan University School of Medicine, Seoul, Republic of Korea				0000-0003-2139-0522	No conflict of interest	Jung-Seok Choi has received a grant from the Korea Mental Health R&D Project, funded by the Ministry of Health & Welfare, Republic of Korea (HI22C0404 to Jung-Seok Choi), and from the National Research Foundation of Korea (2021R1F1A1046081 to Jung-Seok Choi).
Mariano		Chóliz	Psychology School, University of Valencia, Valencia, Spain				0000-0002-4324-2603	No conflict of interest	N/A
Darren	R.	Christensen	Faculty of Health Sciences, University of Lethbridge, Lethbridge, Alberta, Canada	Alberta Gambling Research Institute, University of Lethbridge, Lethbridge, Alberta, Canada			0000-0002-6410-8032	No conflict of interest	N/A

Jenny		Cisneros Örnberg	Department of Public Health Sciences, Centre for Social Research on Alcohol and Drugs (SoRAD), Stockholm University, Stockholm, Sweden				0000-0003-2702-6553	No conflict of interest	Funding for Jenny Cisneros Örnberg was provided within the frame of the Swedish program grant “Responding to and Reducing Gambling Problems – Studies in Help-seeking, Measurement, Comorbidity and Policy Impacts” (REGAPS), financed by the Swedish Research Council for Health, Working Life and Welfare (Forte), grant number 2016-07091.
Luke		Clark	Centre for Gambling Research at UBC, Department of Psychology, University of British Columbia, Vancouver, B.C., Canada	Djavad Mowafaghian Centre for Brain Health, University of British Columbia, Vancouver, British Columbia, Canada			0000-0003-1103-2422	Luke Clark is the Director of the Centre for Gambling Research at UBC, which is supported by funding from the Province of British Columbia and the British Columbia Lottery Corporation (BCLC), a Canadian Crown Corporation. The Province of BC government and the BCLC had no role in the preparation of this article and impose no constraints on publishing. Luke Clark has received travel expenses from Scientific Affairs (Germany), the International Center for Responsible Gaming (US), and the Institut fur Glucksspiel und Gesellschaft (Germany). He has received fees for academic services and consultancy from Scientific Affairs (Germany), the International Center for Responsible Gaming (US), GambleAware (UK), Gambling Research Australia, and Gambling Research Exchange Ontario (Canada). He has been remunerated for legal consultancy by the BCLC. He has not received any further direct or indirect payments from the gambling industry or groups substantially funded by gambling. Luke Clark receives an honorarium for his role as Co-Editor-in-Chief for International Gambling Studies from Taylor & Francis, and he has received royalties from Cambridge Cognition Ltd. relating to neurocognitive testing.	N/A
Irene		Cogliati Dezza	Center for Research in Cognition & Neurosciences, ULB Neuroscience Institute, Université Libre de Bruxelles, Avenue				0000-0002-1212-4751	No conflict of interest	N/A

			Franklin Roosevelt 50, Belgium						
David		Columb	Department of Child and Adolescent Psychiatry, Lucena Clinic Services, Dublin, Ireland	UCD School of Medicine and Medical Specialties, University College Dublin, Dublin, Ireland			0000-0003-3933-9389	No conflict of interest	N/A
Olivier		Corbeil	Faculty of Pharmacy, Université Laval, Québec City, QC, Canada	Quebec Mental Health University Institute, Québec City, QC, CAN			0000-0002-4192-853X	No conflict of interest	Olivier Corbeil has received a fellowship award from the Canadian Institutes of Health Research (#202210MFE-491926-64860).
Aurélien		Cornil	Adaptation, Resilience and Change (ARCh), Faculty of Psychology, Speech and language therapy and Education (FPLSE), ULiège, Belgium	Psychological Sciences Research Institute (IPSY), UCLouvain, Belgium	Laboratoire de Psychologie des Pays de la Loire (LPPL), Nantes Université, France		0000-0002-4961-0681	No conflict of interest	N/A
Pinhas		Dannon	Head Psychiatry Ward, Jerusalem, Israel	Hebrew University of Jerusalem , Jerusalem Israel			0000-0002-4623-8140	No conflict of interest	N/A
Christal	N.	Davis	Ralph H. Johnson VA Medical Center, Charleston, SC, United States	Department of Psychiatry, College of Medicine, Medical University of South Carolina, Charleston, SC, United States			0000-0003-3974-5598	No conflict of interest	N/A
Paul		Delfabbro	University of Adelaide, Adelaide, South Australia, Australia				0000-002-0466-5611	Paul Delfabbro has done paid consultancy work for government and industry groups.	N/A
Jeffrey	Lee	Derevensky	Professor Emeritus, McGill University, Montreal, Quebec, Canada	Director, International Centre for Youth Gambling Problems and High Risk Behaviors, Montreal, quebec, Canada			0000-0001-6947-2987	No conflict of interest	N/A
Gaëtan		Devos	UCLouvain, Research Institute for Psychological Sciences, Louvain-la-Neuve, Belgium	Grand Hôpital de Charleroi (GHdC), Charleroi, Belgium	Scientific Research and Publication Cell (CRPS), Namur, Belgium	Service Universitaire D'Addictologie de Lyon (SUAL), CH le Vinatier, F-69500, Bron, France	0000-0002-1753-8494	No conflict of interest	N/A
Mike	J.	Dixon	Department of Psychology, University of Waterloo, Waterloo, Ontario, Canada				0000-0003-2629-939X	No conflict of interest	Mike J. Dixon's research is funded by the Natural Sciences and Engineering Research Council of Canada
Maria Anna		Donati	NEUROFARBA Department, University of Florence, Florence, Italy				0000-0001-7638-9137	No conflict of interest	N/A

Nicki	A.	Dowling	School of Psychology, Deakin University, Burwood, Australia				0000-0001-8592-2407	In the last three years, Nicki A. Dowling has received research and consultancy funding from multiple sources, including via hypothecated taxes from gambling revenue. Nicki A. Dowling has received research funding from the Victorian Responsible Gambling Foundation, New South Wales Office of Responsible Gambling, Svenska Spel's Independent Research Council, Health Research Council of New Zealand, and New Zealand Ministry of Health. She has not knowingly received research or consultancy funding from the gambling, tobacco, or alcohol industries or any industry-sponsored organisation.	N/A
Magali		Dufour	Département de psychologie, Université du Québec à Montréal, Montréal, Canada				0000-0001-7288-937X	No conflict of interest	Magali Dufour receives funding from the Human Social Research Council (CRSH), Quebec Ministry of Health and Social Services (MSSS), Fonds de recherche du Québec - Société et culture (FRQSC)
Simon		Dymond	School of Psychology, Swansea University, Swansea, United Kingdom.	Department of Psychology, Reykjavík University, Reykjavík, Iceland			0000-0003-1319-4492	Simon Dymond is Director of the GREAT Network Wales, which is funded by Welsh Government through Health and Care Research Wales (HCRW). The views expressed are those of the author and not necessarily those of HCRW or Welsh Government. Simon Dymond is a founding member of the Academic Forum for the Study of Gambling (AFSG) and sits on the Executive Committee (Outreach and Membership Co-Chair) for which he receives an honourarium. He is a director and shareholder in Soteria Global Services, a risk management business with a focus on gambling harm.	Simon Dymond's group has received funding from multiple sources including GambleAware, Gambling Commission (regulatory settlements fund), International Center for Responsible Gaming, Greo, and the Bristol Hub for Gambling Harms Research.
Enrique		Echeburúa	Facultad de Psicología, Universidad del País Vasco (UPV/EHU), San Sebastián, Spain				0000-0001-7654-0781	No conflict of interest	N/A
Boris		Egloff	Department of Psychology, Johannes Gutenberg-University Mainz, Mainz, Germany				0000-0002-5736-9912	No conflict of interest	N/A

Jennifer	D.	Ellis	Department of Psychiatry & Behavioral Sciences, Johns Hopkins School of Medicine, Baltimore, MD, USA				0000-0002-8156-6772	No conflict of interest	N/A
Tara		Elton-Marshall	School of Epidemiology and Public Health, Faculty of Medicine, University of Ottawa, Ottawa, Ontario, Canada	Institute for Mental Health Policy Research, Centre for Addiction and Mental Health, Toronto, Ontario, Canada			0000-0002-1674-8588	No conflict of interest	N/A
Ana		Estévez	Facultad de Ciencias de la Salud, University of Deusto, Bilbao, Spain				0000-0003-0314-7086	No conflict of interest	N/A
Repairer		Etuk	Department of Psychology, University of Nevada, Las Vegas, NV, USA				0000-0003-0648-8687	No conflict of interest	Repairer Etuk has previously received grant funding from the International Gaming Institute, the Nevada Council on Problem Gambling, and the International Center for Responsible Gaming. These institutes had no influence on the answers given for this project.
Shirley		Fecteau	CERVO Brain Research Centre, Centre intégré universitaire en santé et services sociaux de la Capitale-Nationale, Department of Psychiatry and Neurosciences, Faculty of Medicine, Université Laval, Quebec City, Quebec, Canada				0000-0002-9781-4451	No conflict of interest	N/A
Fernando		Fernández-Aranda	Clinical Psychology Department, University Hospital of Bellvitge-IDIBELL, Barcelona, Spain	Department of Clinical Sciences, School of Medicine and Health Sciences, University of Barcelona, Barcelona, Spain	CIBER Fitopatología de la Obesidad y Nutrición, ISCIII, Barcelona, Spain		0000-0002-2968-9898	Fernando Fernández-Aranda received consultancy honoraria from Novo Nordisk.	Fernando Fernández-Aranda's contribution to this work was partially funded by Ministerio de Ciencia e Innovación (PDI2021-124887OB-I00), Instituto de Salud Carlos III (ISCIII) (Exp: FIS22053—Ref: DTS22/00072), European Union's Horizon 2020 research and innovation program under Grant agreement no. 101080219 (eprObes), and cofounded by FEDER (funds/European Regional Development Fund (ERDF), a way to build Europe). CIBERObn is an initiative of ISCIII. FFA is partially supported by ICREA under the ICREA Academia programme.

Naomi	Anne	Fineberg	School of Life and Medical Sciences, University of Hertfordshire, Hatfield, UK	Hertfordshire Partnership University NHS Foundation Trust, Hatfield, UK			0000-0003-1158-6900	Naomi Anne Fineberg's department has received funding from COST Action, Horizon Europe, the UKRI and the Swiss State Secretariat for Education, Research and Innovation for research into behavioural addiction. Naomi Anne Fineberg reports additional relationships with the National Institute of Health Research, Orchard and UK Research and Innovation (UKRI) that includes: funding grants to her department; the Global Mental Health Academy that includes: speaking and lecture fees; the European College of Neuropsychopharmacology, British Association for Psychopharmacology, World Psychiatric Association, International College of Neuropsychopharmacology and Royal College of Psychiatrists that includes: travel reimbursement; Orchard and the European College of Neuropsychopharmacology that includes: board membership; Children and Screens that includes honorarium for lecturing, and research support in kind from Compass Pathways.	Naomi Anne Fineberg's contribution to this work was in part funded by the European Union (BootStRaP project; grant number: 10108238), UK Research and Innovation program [project number: 10075008] and the Swiss State Secretariat for Education, Research and Innovation (SERI).
Gabriele		Fischer	Center of Public Health, Medical University Vienna, Vienna, Austria				0000-0001-5602-929X	Gabriele Fischer received speaking honorarium from industry from Takeda, Camurus, Novonordisk.	N/A
Mal		Flack	Researchers in Behavioural Addictions, Alcohol, and Drugs (BAAD), Faculty of Health, Charles Darwin University, Darwin, Northern Territory, Australia				0000-0002-0181-3631	No conflict of interest	N/A
David		Forsström	Competence Centre for psychotherapy, Centre for psychiatry research, Department of Clinical Neuroscience, Karolinska institute, Stockholm, Sweden				0000-0003-2004-2366	No conflict of interest	David Forsström has received grants from Svenska Spel's independent research Council, University of Bergen and the Swedish Public Health Agency
Rebecca	G.	Fortgang	Department of Psychiatry, Massachusetts General Hospital, Boston, MA, USA	Department of Psychology, Harvard University, Cambridge, MA, USA			0000-0001-5388-3386	No conflict of interest	Rebecca G. Fortgang's work was supported by the NIMH (K23MH132766-01).

Ingmar	Hubert Anton	Franken	Center for Substance use and Addiction Research (CESAR, Erasmus University Rotterdam), Rotterdam, the Netherlands				0000-0002-7853-2694	No conflict of interest	N/A
Fabio		Frisone	Department of Psychology, Humane Technology Lab, Catholic University of the Sacred Heart, Milan, Italy				0000-0002-1766-831X	No conflict of interest	N/A
Johannes		Fuss	Institute of Forensic Psychiatry and Sex Research, Center for Translational Neuro- and Behavioral Sciences, University of Duisburg-Essen, Essen, Germany				0000-0003-0445-5021	No conflict of interest	N/A
Sally	Melissa	Gainsbury	School of Psychology, Brain and Mind Centre, University of Sydney, Sydney, NSW, Australia				0000-0002-9641-5838	Sally Melissa Gainsbury has received research funding from Entain, Sportsbet, Aristocrat, the University of Sydney, Australian Leisure and Hospitality Group, International Center for Responsible Gaming, Star Entertainment, Betcloud, Behavioural Insights Team, GambleAware, KPMG, GREO, QBE, Australian Cricketers Association, Senet, Norths Collective, Washington State Council, Leagues Clubs Australia.	N/A
Belle		Gavriel-Fried	School of Social Work, Tel Aviv University,Ramat Aviv, Tel Aviv, Israel				0000-0001-5528-5339	No conflict of interest	Belle Gavriel-Fried recieved grants from the from the Israel National Insurance Institute and the Committee for Independent Studies of the National Lottery of Israel.
Anna	Maria	Giannini	Department of Psychology, Sapienza University of Rome, Rome, Italy				0000-0002-0614-4457	No conflict of interest	N/A
Montserrat		Gómez García	CSMA Hospitalet Benito Menni CASM, Barcelona, Spain	IL3-UB, Barcelona, Spain			0009-0001-3281-6165	No conflict of interest	N/A
Joaquín		González-Cabrera	Instituto de Transferencia e Investigación (ITEI). Universidad Internacional de La Rioja (UNIR), Logroño, La Rioja (Spain).				0000-0003-2865-3428	No conflict of interest	Joaquín González-Cabrera has received funds from Ministerio de Consumo (Spain) for a study on loot boxes, the Programa Estatal de Investigación orientada a los retos de la Sociedad (Spanish Ministry of Science), and currently receive them for research projects at Universidad Internacional de La Rioja (UNIR, Spain)

Adam	S.	Goodie	Department of Psychology, University of Georgia, Athens, GA, USA				0009-0003-2900-1904	No conflict of interest	N/A
Alessio		Gori	University of Florence, Florence, Italy				0000-0002-6867-2319	No conflict of interest	N/A
Anna	E.	Goudriaan	Department of Psychiatry, Amsterdam UMC, University of Amsterdam, Amsterdam, The Netherlands	Arkin, Department of Research, Amsterdam, The Netherlands	Amsterdam Institute for Addiction Research, Jellinek TOPGGz, Amsterdam, The Netherlands		0000-0001-8670-9384	No conflict of interest	Anna E. Goudriaan received funding from The Netherlands Organisation for Health Research and Development (VIDI grant 91713354)
Marie		Grall-Bronnec	Nantes Université, CHU Nantes, UIC Psychiatrie et Santé Mentale, Nantes, France	Nantes Université, Univ Tours, CHU Nantes, INSERM, MethodS in Patients centered outcomes and HEalth ResEarch, SPHERE, Nantes, France			0000-0003-0722-7243	Marie Grall-Bronnec declares that the Endowment Fund of the University Hospital of Nantes received funding from the gambling industry (FDJ and PMU) as part of the implementation of the obligation to finance scientific studies on gambling and related addictive disorders (Law n° 2010-476 of May 12th modified, art. 3). This funding does not concern the present study, has never had any influence on the present work and scientific independence, objectivity and impartiality is guaranteed.	N/A
Roser		Granero	Departament de Psicobiologia i Metodologia. Universitat Autònoma de Barcelona, Barcelona. Spain				0000-0001-6308-3198	No conflict of interest	N/A
Mark	D.	Griffiths	International Gaming Research Unit, Psychology Department, Nottingham Trent University, UK					Mark D. Griffiths has received research funding from Norsk Tipping (the gambling operator owned by the Norwegian government). Mark D. Griffiths has received funding for a number of research projects in the area of gambling education for young people, social responsibility in gambling and gambling treatment from Gamble Aware (formerly the Responsibility in Gambling Trust), a charitable body which funds its research program based on donations from the gambling industry. Mark D. Griffiths undertakes consultancy for various gambling companies in the area of player protection and social responsibility in gambling.	N/A

Joshua	B.	Grubbs	Department of Psychology, University of New Mexico, New Mexico, USA	Center on Alcohol, Substance use, And Addictions			0000-0002-2642-1351	No conflict of interest	Joshua B. Grubbs receives funding from the Kindbridge Research Institute, the International Center for Responsible Gaming, and the Problem Gambling Network of Ohio,
Tara		Hahmann	MAP Centre for Urban Health Solutions, Toronto, Ontario, Canada				0000-0001-7374-6979	No conflict of interest	N/A
Anders		Håkansson	Lund University, Faculty of Medicine, Department of Clinical Sciences Lund, Lund, Sweden	Region Skåne, Malmö Addiction Center, Competence Center Addiction, Malmö, Sweden			0000-0002-5800-8975	Anders Håkansson has funding from the state-owned gambling operator of Sweden, AB Svenska Spel, and from its research council. Funding from the research council of the state-owned alcohol monopoly of Sweden, Systembolaget.	N/A
Brian	J.	Hall	Center for Global Health Equity, New York University, Shanghai, China				0000-0001-9358-2377	No conflict of interest	N/A
Juho		Hamari	Gamification Group, Faculty of Information Technology and Communication Sciences, Tampere University, Tempere, Finland				0000-0002-6573-588X	No conflict of interest	Juho Hamari has received funding from Kone Foundation (202008478), Academy of Finland (337653)
Wei		Hao	Department of Psychiatry & Mental Health Institute of the Second Xiangya Hospital, Central South University, Changsha, Hunan, China	National Clinical Research Center on Mental Disorders & National Technology Institute on Mental Disorders, Changsha, Hunan, China			0000-0002-3392-1653	No conflict of interest	N/A
Tobias		Hayer	Institute of Public Health and Nursing Research, Department for Health and Society, University of Bremen, Germany				0000-0002-0225-8764	No conflict of interest	Tobias Hayer has received financial support as principal investigator from the Federal Ministry of Health, various German federal states, the Joint State Gambling Authority (national gambling regulator), the Deutsche Forschungsgemeinschaft (DFG, German Research Foundation) and the Legal Committee of the Deutscher Lotto- und Totoblock (Germany's state lottery association)

Robert	Michael	Heirene	Brain & Mind Centre, School of Psychology, University of Sydney, Sydney, Australia				0000-0002-5508-7102	Robert Michael Heirene has worked on a project funded by Responsible Wagering Australia (a representative body of Australian online wagering operators; University of Sydney, 2019-2021) and as an independent, sub-contracted statistical consultant for PRET Solutions Inc on a commissioned project (funded by the Australian Casino operator Crown; 2023). In 2023, Robert Michael Heirene was a co-investigator on a successful grant from the International Centre for Responsible Gaming, which now partially supports his Research Fellow role at the University of Sydney	N/A
Nerilee		Hing	Experimental Gambling Research Laboratory, School of Health, Medical and Applied Sciences, Central Queensland University, Bundaberg, Australia				0000-0002-2150-9784	No conflict of interest	Nerilee Hing has received funding from numerous government sources: Gambling Research Australia, the Victorian Responsible Gambling Foundation, the NSW Responsible Gambling Fund and NSW Office of Responsible Gambling, the New Zealand Ministry of Health, the South Australian Office for Problem Gambling, the ACT Gaming and Racing Commission, Australia's National Research Organisation for Women's Safety, and the Australian Media and Communications Authority. I have also been subcontracted to assist with research projects conducted by Engine Consulting, First Person Consulting, and the First Nations Foundation.
Niklas		Hopfgartner	Institute of Interactive Systems and Data Science, Graz University of Technology, Graz, Austria	neccton GmbH, Müllendorf, Austria			0000-0002-4672-7956	No conflict of interest	N/A
Tristan	Joseph	Hynes	Department of Psychology, University of Cambridge, Cambridge UK				0000-0002-9400-2458	No conflict of interest	N/A

Richard	J. E.	James	School of Psychology, University of Nottingham, Nottingham, Nottinghamshire, United Kingdom				0000-0002-6644-7011	Richard J. E. James is currently principal investigator on projects funded by Gambling Research Exchange Ontario (GREO) and the Academic Forum for the Study of Gambling (AFSG). The funds for these projects come from regulatory settlements levied by the UK Gambling Commission. Richard J. E. James was previously a co-investigator on a seed grant from the International Center for Responsible Gaming, which is funded by donations from the gambling industry and administered by a scientific panel.	N/A
Paula		Jauregui	University of Deusto, Bilbao, Spain				0000-0002-9706-0274	No conflict of interest	N/A
Emilien		Jeannot	Centre du jeu excessif, Addiction medicine, Lausanne University Hospital and University of Lausanne, Lausanne, Switzerland	Faculty of Medicine, Institute of Global Health,University of geneva , Geneva, Switzerland			0000-0002-6625-3575	No conflict of interest	N/A
Andrew	S.	Kayser	Department of Neurology, UC San Francisco, USA	Department of Veterans Affairs, San Francisco, USA	Helen Wills Neuroscience Institute, UC Berkeley, Berkeley, CA, USA		0000-0001-5102-2136	Andrew S. Kayser has received consulting fees from Boehringer-Ingelheim.	N/A
Yasser		Khazaal	Addiction medicine, Lausanne University Hospital and Lausanne University, Lausanne, Switzerland	Montreal University, Montréal, Canada			0000-0002-8549-6599	No conflict of interest	N/A
Hyoun	S.	Kim	Department of Psychology, Toronto Metropolitan University, Toronto, ON, Canada	University of Ottawa Institute of Mental Health Research at The Royal, Ottawa, ON, Canada			0000-0002-0804-0256	No conflict of interest	N/A
Serena		King	Department of Psychology, Hamline University, Saint Paul, MN , United States	University of Minnesota, Center for Twin and Family Research, Affiliate Research Scientist, MN , United States			0000-0002-8640-385X	No conflict of interest	N/A
Keiji		Kobara	Osaka University of Commerce, Osaka, Japan				0000-0001-7066-8266	No conflict of interest	N/A
Komathi		Kolandai	Public Policy Institute, University of Auckland, Auckland, New Zealand	COMPASS Research Centre, University of Auckland, Auckland, New Zealand			0000-0002-1633-3067	No conflict of interest	N/A
Toula		Kourgiantakis	École de travail social et de criminologie, Université Laval, Québec, QC Canada				0000-0002-2491-2595	No conflict of interest	N/A

Ildikó		Kovács	Department of Psychiatry, Albert Szent-Györgyi Medical School, University of Szeged, Szeged, Hungary				0000-0003-4215-8351	No conflict of interest	N/A
Shane	W.	Kraus	Department of Psychology, University of Nevada, Las Vegas, Nevada, USA				0000-0002-0404-9480	No conflict of interest	N/A
Ludwig		Kraus	Department of Public Health Sciences, Centre for Social Research on Alcohol and Drugs, Stockholm University, Stockholm, Sweden	Institute of Psychology, ELTE Eötvös Loránd University, 1Budapest, Hungary	Centre of Interdisciplinary Addiction Research (ZIS), Department of Psychiatry and Psychotherapy, University Medical Centre Hamburg-Eppendorf, Hamburg, Germany		0000-0001-7282-0217	No conflict of interest	Ludwig Kraus has received funding from The Swedish programme grant 'Responding to and Reducing Gambling Problems – Studies in Help-seeking, Measurement, Comorbidity and Policy Impacts' financed by the Swedish Research Council for Health, Working Life and Welfare (Forte), grant number 2016–07091
Søren		Kristiansen	Department of Sociology and Social Work, Aalborg University, Denmark				0000-0001-7211-8882	No conflict of interest	N/A
Daria	J.	Kuss	Nottingham Trent University, Nottingham, UK				0000-0001-8917-782X	No conflict of interest	N/A
Jason		Landon	Department of Psychology and Neuroscience, Auckland University of Technology, Auckland, New Zealand				0000-0002-3595-7430	No conflict of interest	N/A
Debi	A.	LaPlante	Division on Addiction, Cambridge Health Alliance, Malden, MA, USA	Harvard Medical School, Department of Psychiatry, Boston, MA, USA			0000-0001-5418-5504	Debi A. LaPlante has served as a paid grant reviewer for the International Center for Responsible Gaming (ICRG), received travel funds, speaker honoraria, and a scientific achievement award from the ICRG, has received speaker honoraria and travel support from the National Collegiate Athletic Association received publication royalty fees from the American Psychological Association, and received course royalty fees from the Harvard Medical School Department of Continuing Education. Debi A. LaPlante is a non-paid member of the New Hampshire Council for Responsible Gambling.	The Division on Addiction at Cambridge Health Alliance currently receives funding from DraftKings, Inc., a sports betting and gaming company; Entain PLC (formally GVC Holdings PLC), a sports betting and gambling company; EPIC Risk Management; Foundation for Advancing Alcohol Responsibility, a not-for-profit organization founded and funded by a group of distillers; International Center for Responsible Gaming; Massachusetts Department of Public Health, Office of Problem Gambling Services via Health Resources in Action; National Academy of Medicine; and National Institutes of Health (National Institutes of General

									Medical Sciences, and Drug Abuse, and Mental Health) via The Healing Lodge of the Seven Nations.
Bernard		Le Foll	Translational Addiction Research Laboratory, Centre for Addiction and Mental Health, Toronto, Canada	University of Toronto, Toronto, Canada	Waypoint Centre for Mental Health Care, Penetanguishene, Canada		0000-0002-6406-4973	No conflict of interest	Bernard Le Foll is supported by CAMH, Waypoint Centre for Mental Health Care, a clinician-scientist award from the department of Family and Community Medicine of the University of Toronto and a Chair in Addiction Psychiatry from the department of Psychiatry of University of Toronto.
David	M.	Ledgerwood	Department of Psychiatry and Behavioral Neurosciences, Wayne State University, Detroit, MI, USA				0000-0003-3122-0199	David M. Ledgerwood has received research grant funding from the Manitoba Liquor & Lotteries Corporation. In the past 5 years he has also received speaking or consulting fees from Yale University, UCLA, The Evergreen Council on Problem Gambling, Iowa Department of Health and Human Services, Responsible Gambling Association of New Mexico, Morneau-Shepell, Oklahoma Association on Problem and Compulsive Gambling, and State of Arizona Department of Gambling.	N/A
Bonnie	K.	Lee	Faculty of Health Sciences, University of Lethbridge, Lethbridge, Canada				0000-0002-6601-3416	No conflict of interest	N/A
Bernadeta		Lelonek-Kuleta	Department of Psychology, The John Paul II Catholic University of Lublin, Lublin, Poland				0000-0002-7844-3667	No conflict of interest	N/A
En		Li	School of Business and Law, Central Queensland University, Rockhampton, QLD, Australia				0000-0001-8732-4988	No conflict of interest	N/A
Kalle		Lind	Finnish Institute for Health and Welfare, Department of Public Health and Welfare, Helsinki, Finland				0000-0003-0238-3837	No conflict of interest	The daily work and research projects of Kalle Lind are funded by the Ministry of Social Affairs and Health, Finland, under the objectives of §52 Appropriation of the Lotteries Act

Philip		Lindner	Centre for Psychiatry Research, Department of Clinical Neuroscience, Karolinska Institutet, & Stockholm Health Care Services, Region Stockholm, Stockholm, Sweden				0000-0002-3061-501X	Philip Lindner reports several past and ongoing academia-industry collaborations with gambling providers (including commissioned research), all under the condition of full academic freedom, and has not personally received any money from the gambling industry, nor has any other financial ties.	N/A
Jakob		Linnet	Clinic on Gambling- and Binge Eating Disorder, Department of Occupational and Environmental Medicine, Odense University Hospital, Denmark.				0009-0003-5899-2444	No conflict of interest	N/A
Suzanne		Lischer	Lucerne University of Applied Sciences and Arts, Luzern, Switzerland				0000-0002-5732-8627	No conflict of interest	N/A
Joanne		Lloyd	School of Psychology, University of Wolverhampton, West Midlands, UK				0000-0003-3891-7247	Joanne Lloyd has received research and consultancy funding (via the University of Wolverhampton) from GambleAware within the past 5 years.	N/A
Helen	Mary	Lloyd	School of Psychology, University of Plymouth, Plymouth, Devon, UK				0000-0002-2916-1874	No conflict of interest	N/A
Christine		Lochner	SA MRC Unit on Risk and Resilience in Mental Disorders, Department of Psychiatry, Stellenbosch University, Cape Town, South Africa				0000-0002-4766-3704	No conflict of interest	N/A
Hibai		Lopez-Gonzalez	Department of Library, Information Science, and Communication, University of Barcelona, Barcelona, Spain.				0000-0003-1249-2623	No conflict of interest	N/A
Valentina		Lorenzetti	Neuroscience of Addiction and Mental Health Program, Healthy Brain and Mind Research Centre, School of Behavioural & Health Sciences, Faculty of Health Sciences, Australian Catholic University, Fitzroy, Australia				0000-0002-5917-7068	No conflict of interest	Valentina Lorenzetti is supported by an Al and Val Rosenstrauss Research Fellowship (2022-2026), and by a National Health Medical Research Council Investigator Grant ID:2016833 (2023-2027).

Eric	R.	Louderback	Division on Addiction, Cambridge Health Alliance, Malden, MA, USA	Harvard Medical School, Department of Psychiatry, Boston, MA, USA			0000-0002-9754-9790	During the past five years, Eric R. Louderback has provided paid consulting services on player safety programs for Premier Lotteries Ireland, and has received travel reimbursement and speaker honoraria fees from the International Center for Responsible Gaming (ICRG). He has also received travel reimbursement and speaker honoraria fees from the Responsible Gaming Association of New Mexico. Dr. Louderback is a researcher at the Division on Addiction, which currently receives funding from the Cambridge Community Foundation; DraftKings, Inc., a sports betting and gaming company; ESPN via The University of Nevada, Las Vegas International Gaming Institute; Foundation for Advancing Alcohol Responsibility, a not-for-profit organization founded and funded by a group of distillers; Greater Boston Council on Problem Gambling; International Center for Responsible Gaming; Massachusetts Department of Public Health, Office of Problem Gambling Services via Health Resources in Action; National Council on Problem Gambling; and National Institutes of Health (National Institutes of General Medical Sciences, and Drug Abuse, and Mental Health) via The Healing Lodge of the Seven Nations. During the past five years, the Division on Addiction has also received funding from the City of Seattle; Entain PLC (formally GVC Holdings PLC), a sports betting and gambling company; EPIC Risk Management; Integrated Centre on Addiction Prevention and Treatment of the Tung Wah Group of Hospitals, Hong Kong; Kimley-Horn; Massachusetts Department of Public Health, Bureau of Substance Addiction Services; Massachusetts Department of Public Health, Bureau of Substance Addiction Services via St. Francis House; Massachusetts Department of Public Health, Office of Problem Gambling Services via Health Resources in Action; the Massachusetts Gaming Commission, Commonwealth of Massachusetts;	Eric R. Louderback's funders had no input or influence during any stage of the research process for the present study.

									MGM Resorts International via the University of Nevada, Las Vegas; National Academy of Medicine; Substance Abuse and Mental Health Services Administration via the Addiction Treatment Center of New England; and Substance Abuse and Mental Health Services Administration via the Gavin Foundation.	
Johanna	Kathari na	Loy	Child and adolescent psychiatry, University hospital of Cologne, Germany				0000-0003-1740-7002		No conflict of interest	N/A
Amandine		Luquiens	University of Montpellier, Universitary hospital of Nîmes, Nîmes, France	CESP, Univ. Paris-Sud, UVSQ, INSERM, Université Paris Saclay, Villejuif, France			0000-0002-9402-442X		Amandine Luquiens has data sharing agreement with Winamax and FDJ and PMU. Scientific independence towards gambling industry operators is warranted. There were no constraints on publishing.	Amandine Luquiens was the recipient of a grant regulated by a public organism "French observatory of addictive behaviors- OFDT" and constraining all French monopolistic gambling service providers to redistribute 0.002% of stakes on their platforms to academic research. The gambling service provider implied in that grant was the "Paris Mutuel Urbain" (PMU). Independency of the research with no constraint on the protocol, the analysis and the publication were guaranteed by a strict convention between

									universities, hospitals and the PMU.
Joseph		Macey	Centre of Excellence in Game Culture Studies, University of Turku, Finland	Gamification Group, Tampere University, Finland.			0000-0002-9770-739X	No conflict of interest	Joseph Macey has received funding from the Academy of Finland through the Centre of Excellence in Game Cultures (Grant 353268)
Juan	Manuel	Machimbarrena	Department of Clinical Psychology and Health Psychology and Research Methodology, Faculty of Psychology, University of the Basque Country (UPV/EHU), San Sebastián, Spain				0000-0002-5506-3661	No conflict of interest	N/A
Laura		Macía Guerrero	Psychology Department, School of Health Sciences, University of Deusto, Bilbao, Spain				0000-0002-3290-9185	No conflict of interest	N/A
Núria		Mallorquí-Bagué	Department of Psychology, University of Girona (UdG), Girona, Spain				0000-0003-1434-3162	No conflict of interest	Núria Mallorquí-Bagué is appointed under the Serra Húnter Programme, a Catalan Government initiative to promote academic excellence."
Viktor		Månsson	Region Dalarna, Division of Psychiatry, Sweden	Center for Psychiatry Research, Department of Clinical Neuroscience, Karolinska Institutet, Stockholm, Sweden	Center for Clinical Research Dalarna, Uppsala University, Uppsala, Sweden		0000-0001-8954-6875	No conflict of interest	N/A
Loredana	A.	Marchica	Department of Psychology, Montreal Children's Hospital, McGill University Health Center, Montreal, Quebec, Canada				0000-0002-5321-0058	No conflict of interest	N/A
Emanuela		Mari	Department of Psychology, Sapienza University of Rome, Rome, Italy				0000-0003-2367-3139	No conflict of interest	N/A
Simon		Marmet	School of Social Work, University of Applied Sciences and Arts Northwestern Switzerland, Olten, Switzerland				0000-0002-9060-8567	No conflict of interest	N/A

Giovanni		Martinotti	Department of Neuroscience, Imaging, Clinical Sciences, University G.d'Annunzio, Chieti-Pescara, Chieti, Italy				0000-0002-7292-2341	No conflict of interest	N/A
Flora	I.	Matheson	MAP Centre for Urban Health Solutions, St. Michael's Hospital, Unity Health Toronto, Toronto, Ontario, Canada	Dalla Lana School of Public Health, University of Toronto, Toronto, Ontario, Canada	Centre for Criminology & Sociolegal Studies, University of Toronto, Toronto, Ontario, Canada		0000-0003-0965-1048	No conflict of interest	Dr. Matheson is supported by an Endowed Chair in Homelessness, Housing and Health, a joint Hospital-University Endowed Chair between the University of Toronto, the St. Michael's Hospital, and the St. Michael's Hospital Foundation.
Sachio		Matsushita	Kurihama Medical and Addiction Center, Yokosuka, Kanagawa, Japan				0000-0002-2439-7984	No conflict of interest	N/A
André	J.	McDonald	Peter Boris Centre for Addictions Research, McMaster University & St. Joseph's Healthcare Hamilton, Hamilton, ON, Canada				0000-0003-1734-5067	No conflict of interest	N/A
Daniel	S.	Mcgrath	Department of Psychology, University of Calgary, Calgary, Canada				0000-0002-2772-942X	No conflict of interest	N/A
Jose		Menchon	Department of Psychiatry. Bellvitge University Hospital-IDIBELL, Barcelona, Spain	Department of Clinical Sciences. Faculty of Medicine and Health Sciences. University of Barcelona., Barcelona, Spain	CIBERSAM, Barcelona, Spain		0000-0002-6231-6524	No conflict of interest	N/A
Stephanie	S.	Merkouris	School of Psychology, Deakin University, Geelong, Australia				0000-0001-9037-6121	In the last 3 years, Stephanie S. Merkouris has received research funding from multiple sources, including the Victorian Responsible Gambling Foundation, New South Wales Office of Responsible Gambling, Health Research Council of New Zealand, and New Zealand Ministry of Health. Stephanie S. Merkouris has been the recipient of a New South Wales Office of Responsible Gambling Postdoctoral Fellowship. She has not knowingly received research funding from the gambling, tobacco or alcohol industries or industry-sponsored organisation.	N/A
Gemma		Mestre-Bach	Universidad Internacional de La Rioja, La Rioja, Spain				0000-0001-5345-0484	No conflict of interest	GMB and this study were supported by the ITEI B23-010 project (Universidad Internacional de La Rioja) and by the Ministerio de Consumo (SUBV23/00024).

Devin	J.	Mills	Texas Tech University, Lubbock, USA	Department of Community, Family, and Addiction Sciences			0000-0003-3421-1650	Devin J. Mills has conducted consultancy projects within the gambling industry aimed at understanding and promoting responsible gaming practices.	N/A
Lorenzo		Moccia	Department of Neuroscience, Section of Psychiatry, Università Cattolica del Sacro Cuore, Rome, Italy	Department of Psychiatry, Fondazione Policlinico Universitario Agostino Gemelli IRCCS, Rome, Italy			0000-0003-3176-6060	No conflict of interest	N/A
Olof		Molander	Department of Clinical Neuroscience, Centre for Psychiatry Research, Karolinska Institutet, Solna, Sweden				0000-0001-5348-051X	No conflict of interest	N/A
Sabrina		Molinaro	Institute of Clinical Physiology, IFC, National Research Council of Italy - CNR, Pisa, Italy				0000-0001-7221-0873	No conflict of interest	N/A
Pedro		Morgado	Life and Health Sciences Research Institute (ICVS), Braga, Portugal	ICVS/3B's, PT Government Associate Laboratory, Braga/Guimarães, Portugal	Clinical Academic Center, Braga, Portugal		0000-0003-3880-3258	Pedro Morgado has received in the past 3 years grants, CME-related honoraria, or consulting fees from Angelini, AstraZeneca, Bial, Biogen, DGS-Portugal, FCT, FLAD, Janssen-Cilag, Gulbenkian Foundation, Lundbeck, Springer Healthcare, Tecnimede, Viatris and 2CA-Braga.	N/A
Franziska		Motka	LMU Munich, Department of Psychology, Clinical Psychology and Psychotherapy, Munich, Germany				0009-0001-2712-0788	No conflict of interest	N/A
Viktor		Mravcik	First Faculty of Medicine, Charles University, Prague, Czech Republic	Spolecnost Podane ruce, Brno, Czech Republic	National Institute of Mental Health, Klecany, the Czech Republic		0000-0001-5062-5744	Viktor Mravcik is Chairman of the Advisory board of the Responsible Gambling project of the Institute for Gambling Regulation facilitating communication between gambling operators, the regulator and the professionals from addiction field.	Viktor Mravcik has received funding from Project OP JAC "Research of Excellence on Digital Technologies and Wellbeing", No. CZ. 02.01.01/00/22_008/0004583.
Lucero		Munguía	Psychology Clinic Unit, University Hospital of Bellvitge-ICS, Barcelona, Spain	Psychoneurobiology of Eating and Addictive Behaviors Group, Neurosciences Program, Bellvitge Biomedical Research Institute (IDIBELL), Barcelona, Spain	CIBER Fisiopatología Obesidad y Nutrición (CIBERobn), Instituto de Salud Carlos III, Barcelona, Spain		0000-0002-9751-810X	No conflict of interest	N/A
W. Spencer		Murch	Department of Sociology and Anthropology, Concordia University, Montreal, Quebec, Canada				0000-0003-2780-3578	W. Spencer Murch previously received training and funding from The Centre for Gambling Research at University of British Columbia, a research laboratory jointly supported by the Government of British	W. Spencer Murch holds a Postdoctoral Fellowship from the Canadian Institutes of Health Research.

								Columbia and the British Columbia Lottery Corporation (BCLC; a Canadian Crown Corporation).	
Juan	F.	Navas	Department of Personality, Assessment, and Clinical Psychology, Universidad Complutense de Madrid, Spain				0000-0002-9521-6642	No conflict of interest	N/A
Sarah	E.	Nelson	Division on Addiction, Cambridge Health Alliance, Malden, MA, USA	Harvard Medical School, Department of Psychiatry, Boston, MA, USA			0000-0001-7967-4910	In the past five years, Sarah E. Nelson has received travel reimbursement and speaker honoraria from the International Center for Responsible Gaming (ICRG) and Responsible Gaming Association of New Mexico, and served as a paid grant reviewer for ICRG. As a researcher at the Division on Addiction, some of Sarah E. Nelson's salary derives from grants and contracts with gambling industry, specified in the Funding statement.	Projects in which Sarah E. Nelson is involved at the Division on Addiction currently receive funding from DraftKings, Inc., a sports betting and gaming company; Entain PLC, a sports betting and gambling company; Foundation for Advancing Alcohol Responsibility, a not-for-profit organization founded and funded by a group of distillers; and the National Institutes of Health.
Laura	Louise	Nicklin	School of Education, University of Wolverhampton, England				0000-0002-6195-9501	No conflict of interest	LLN has worked on research projects (via the University of Wolverhampton) funded by Gamble Aware within the past 5 years.
Giovanna		Nigro	Department of Psychology, University of Campania "Luigi Vanvitelli", Caserta, Italy				0000-0003-3518-2468	No conflict of interest	N/A
Anders		Nilsson	Center for Psychiatry Research, Department of clinical neuroscience, Karolinska Institutet, Stockholm, Sweden				0000-0002-0982-8483	No conflict of interest	N/A
Xavier		Noel	Laboratoire de Psychologie Médicale et d'Addictologie, Faculty of Medicine, Université Libre de Bruxelles (ULB), Brussels, Belgium				0000-0002-4604-8144	Xavier Noel is involved in multiple research projects that focus on studying the phenomenon of loss-chasing by analyzing online gambling data in collaboration with gambling operators.	Xavier Noel is backed by the Fonds de la Recherche Scientifique (F.R.S.-FNRS; https://www.frs-fnrs.be/en/). Moreover, as a research associate overseeing gambling research at the Centre for Behavioral Addiction Research at ULB (Free University of Brussels, ULB), he benefits from research support provided by the Belgian Association of Gaming Operators (BAGO). No honorarium has been accepted. Additionally, Xavier Noël has undertaken various training missions and

									research endeavors for the Belgian Gambling Commission (Minister of Justice).
Lia		Nower	Center for Gambling Studies, Rutgers University School of Social Work, New Brunswick, NJ USA				0000-0002-2497-8957	Lia Nower has been a member of advisory boards or conducted research, training/presentations, grant reviews, and/or consultations funded by US or international, government, or government-related funding agencies, private firms, and industry operators. In the past five years, these include the State of New Jersey, Division of Gaming Enforcement & Division of Mental Health and Addiction Services (U.S.); Ohio Department of Mental Health and Addiction (U.S.); Camelot (United Kingdom); Crown Casino Ltd. (Australia); British Columbia Lottery Corporation (BCLC); Churchill Downs (U.S.); Aristocrat Pty. (Australia), New York Council on Problem Gambling (U.S.); Publiedit (Italy); National Council on Problem Gambling (U.S.).	N/A
Colin		O'Gara	Department of Medicine and Medical Specialties, University College Dublin School of Medicine, Dublin, Ireland	Saint John of God Hospital, Dublin, Ireland			0000-0002-0973-5930	No conflict of interest	N/A
Jane	Elizabe th	Oakes	Flinders University, Adelaide, Australia				0000-0002-3571-2556	No conflict of interest	Jane Elizabeth Oakes receives funding from Gambling Research Australia, Victorian Responsible Gambling Foundation, Flinders University Seeding Grant
Ugo		Pace	Faculty of Human and Social Sciences, University Kore of Enna, ITALY				0000-0003-0168-8153	No conflict of interest	N/A
Stefano		Pallanti	Department of Psychiatry, Montefiore Medical Center, Albert Einstein College of Medicine, New York, USA	Istituto di Neuroscienze, Firenze, Italy			0000-0001-5828-4868	No conflict of interest	Stefano Pallanti has received the R21 grant DA042271
Ståle		Pallesen	Department of Psychosocial Science, University of Bergen, Bergen, Norway	Norwegian Competence Center of Gambling and Gaming Research, University of Bergen, Bergen, Norway			0000-0002-5831-0840	No conflict of interest	N/A

Arpit		Parmar	Department of Psychiatry, All India Institute of Medical Sciences, Bhubaneswar, Odisha, India				0000-0002-0487-0404	No conflict of interest	N/A
Jonathan		Parke	Sophro Limited, Newark Beacon, Newark, Nottinghamshire, United Kingdom NG24 2TN	Sheffield Hallam University, Sheffield, United Kingdom				Over the past five years, Jonathan Parke has received funding for research, education and/or consulting services from Kindred Group Plc, International Game Technology, Allwyn Entertainment, Camelot UK, Betclic Group, Playtech, British Columbia Lottery Corporation, GambleAware and Premier Lotteries Ireland.	N/A
Alberto		Parrado-González	Department of Social, Developmental and Educational Psychology, University of Huelva, Huelva, Spain				0000-0003-3812-9865	No conflict of interest	N/A
Alessia		Passanisi	Faculty of Human and Social Sciences, University of Enna "Kore", Enna, Italy				0000-0001-5140-0256	No conflict of interest	N/A
Raimondo	Maria	Pavarin	University of Bologna, Bologna BO, Italy				0000-0003-1403-3148	No conflict of interest	N/A
José	C.	Perales	Mind, Brain, and Behavior Research Center (CIMCYC), Department of Experimental Psychology, University of Granada, Spain				0000-0001-5163-8811	José C. Perales does not receive any direct or indirect funding by any gambling-related company or institution potentially compromising his total autonomy or independence to conduct research, interpret its results, or make them public.	José C. Perales's research is supported by a R&D grant (MICIU/AEI/10.13039/501100011033), funded by the Spanish Research Agency/Agencia Española de Investigación (Spanish Ministry of Science and Innovation/Ministerio de Ciencia e Innovación), and the European Union (European RegionalDevelopment Fund/Fondo Europeo de Desarrollo Regional - ERDF/FEDER) with reference PID2023-150731NB-I00.
Jan		Peters	Department of Psychology, Biological Psychology, University of Cologne, Cologne, Germany				0000-0002-0195-5357	No conflict of interest	N/A
Rory	A.	Pfund	Tennessee Institute for Gambling Education & Research, The University of Memphis, Memphis, TN, USA				0000-0002-9719-503X	No conflict of interest	Rory A. Pfund has received grant funding from the International Center for Responsible Gaming, the National Institute on Alcohol Abuse and Alcoholism, and the Tennessee Department of Mental Health and Substance Abuse Services.

Kahlil	S.	Philander	School of Hospitality Business Management, Carson College of Business, Washington State University, Everett, WA, USA	School of Psychology, Science Faculty, University of Sydney, Camperdown, NSW, Australia			0000-0002-0747-0772	In the past five years, Kahlil S. Philander received funding for research from the Washington State Gambling Commission, the International Center for Responsible Gambling, the International Center for Gaming Regulation, and Entain (sub-award via the Division on Addiction). He received honoraria from the University of Nevada, Las Vegas, the University of York, and G2E Asia. He received expert witness payments from Crown Resorts Limited, Jones Ward, and Scientific Games. He received consulting payments (directly) from Scientific Affairs, MGM Resorts, British Columbia Lottery Corporation, Seminole Hard Rock Entertainment Inc., Victor Strategies, and Eilers and Krejcik Gaming LLC, the Responsible Gambling Council of Canada, the Division on Addiction, Las Vegas Sands, the Canadian Responsible Gambling Association, the University of Sydney, the Gaming Board for The Bahamas, (indirectly) the Chicago Cubs, Wymac Gaming Solutions, Red Rock Resorts, the West Virginia Lottery, the Indiana Gaming Commission, Little River Casino Resort, and Choctaw Nation of Oklahoma.	N/A
Fulvia		Prever	SUN(N)COOP Women&Gambling Project, Scientific Director, Milano, Italy	Varenna Foundation- President, Milano, Italy			0009-0003-8963-2863	No conflict of interest	N/A
Alessandro		Quaglieri	Faculty of Social and Communication Sciences, "Mercatorum" Universitas, Rome, Italy	Department of Psychology, “Sapienza” University of Rome, Rome, Italy			0000-0003-2341-1876	No conflict of interest	N/A
Jonas		Rafi	Department of Psychology, Stockholm university, Stockholm, Sweden				0000-0001-6292-4389	No conflict of interest	N/A
Carla	J.	Rash	Calhoun Cardiology Center, Departments of Medicine & Psychiatry, UConn School of Medicine, Farmington, CT, USA				0000-0001-9264-6885	Carla J. Rash receives funding from WondrNation, the online gaming arm of the Mashantucket Pequot Tribal Nation.	N/A
Vijay		Rawat	School of Health, Medical, and Applied Sciences, Central Queensland				0000-0003-1539-5116	No conflict of interest	Vijay Rawat has received research funding from Central Queensland University, Gambling Research Australia, the New

			University, Melbourne, Victoria, Australia						South Wales Responsible Gambling Fund, and the Victorian Responsible Gambling Foundation.
Jérémie		Richard	Department of Psychiatry and Behavioral Sciences, Johns Hopkins University School of Medicine, Baltimore, Maryland, USA				0000-0001-9893-1353	No conflict of interest	N/A
Neven		Ricijas	Department of Behavioral Disorders, Faculty of Education and Rehabilitation Sciences, University of Zagreb, Zagreb, Croatia				0000-0001-8107-8448	No conflict of interest	N/A
Amanda		Roberts	School of Psychology, University of Lincoln, Lincoln, UK				0000-0002-2889-9551	Amanda Roberts is Co-Chair of the Executive Committee of the Academic Forum for the Study Gambling (AFSG). Funding for the AFSG is derived from regulatory settlements for socially responsible purposes that are approved by the Gambling Commission, and is administered by Greo. Although funded by Greo, the AFSG operates as an independent entity. She does not have any potential conflicts of interest in relation to gambling or the gambling industry.	N/A
Simone	Nicole	Rodda	Department of Psychology and Neuroscience, Auckland University of Technology, Auckland, New Zealand	School of Psychology, Deakin University, Geelong, Australia			0000-0002-7973-1003	No conflict of interest	N/A
Jim		Rogers	School of Health and Social Care, University of Lincoln, Lincoln, UK				0000-0002-1898-6184	No conflict of interest	For co-chairing the UK based Academic Forum for the Study of Gambling (AFSG) Jim Rogers receives an honorarium from GREO, using funding hypothecated via the Uk Gambling Commission from regulatory settlement monies.
Guyonne		Rogier	Saint Camillus International University of Health and Medical Sciences, Rome, Italy				0000-0001-5320-4478	No conflict of interest	N/A
Sara		Rolando	Eclectica+, Institute for Research and Training, Torino, Italy				0000-0003-1002-2599	No conflict of interest	N/A
Nina		Romanczuk-Seiferth	Department of Psychology, MSB Medical School Berlin, Berlin, Germany				0000-0002-6931-269X	No conflict of interest	N/A

Don		Ross	School of Society, Politics, and Ethics, University College Cork, Cork, Ireland	School of Economics, University of Cape Town, Rondebosch, South Africa	Center for the Economic Analysis of Risk, Robinson College of Business, Georgia State University, Atlanta GA, USA		0000-0003-1813-3111	No conflict of interest	Don Ross has received funding from the Center for the Economic Analysis of Risk, Robinson College of Business, Georgia State University
Hans-Jürgen		Rumpf	Department of Psychiatry and Psychotherapy, University of Lübeck, Lübeck, Germany				0000-0001-6848-920X	No conflict of interest	N/A
Gillian	Erin Hutchison	Russell	Criminal Justice Research Center, Penn State Abington, Abington, PA, USA				0000-0002-4576-6101	No conflict of interest	N/A
Alex	M. T.	Russell	Experimental Gambling Research Laboratory, CQUniversity, Sydney, NSW, Australia				0000-0002-3685-7220	No conflict of interest	In the last five years, Alex M. T. Russell has received funding for research from Gambling Research Australia, the Victorian Responsible Gambling Foundation, the Victorian Department of Justice and Community Safety, the New South Wales Office of Responsible Gambling via the Responsible Gambling Fund, Liquor and Gaming New South Wales, the Australian Capital Territory Gambling and Racing Commission, the Queensland Department of Justice, the Northern Territory Department of Industry, Tourism and Trade, the Australian Communications and Media Authority, the New Zealand Ministry of Health and the South Australian Government. He has also been named on research not related to gambling, funded by Arts Queensland.
Paul		Sacco	University of Maryland-Baltimore, Baltimore, Maryland				0000-0002-2800-9571	No conflict of interest	Paul Sacco has received some funding support from ICRG.
Dominic		Sagoe	Department of Psychosocial Science, University of Bergen, Bergen, Norway	Norwegian Competence Centre for Gambling and Gaming Research, University of Bergen, Bergen, Norway			0000-0002-1902-9378	No conflict of interest	N/A
Anne	H.	Salonen	Research Manager, Finnish Institute for Health and Welfare, Department	Associate Professor, University of Eastern Finland, Faculty of Health Sciences, Finland			0000-0002-4693-0110	No conflict of interest	Daily work and research projects of Anne H. Salonen are funded by the Ministry of Social Affairs and Health, Finland, within the

			of Public Health and Welfare, Finland						objectives of the §52 Appropriation of the Lotteries Act.
Eva		Samuelsson	Department of Social Work, Stockholm University, Stockholm, Sweden	Department of Public Health Sciences, Stockholm University, Stockholm, Sweden			0000-0002-0856-9854	No conflict of interest	Eva Samuelsson’ work was supported by the research program Responding to and Reducing Gambling Problems Studies (REGAPS) funded by Forte
James	Ladell	Sanders	Faculty of Health Sciences, University of Lethbridge, Lethbridge, Alberta, Canada				0000-0001-7398-2031	No conflict of interest	N/A
John	B.	Saunders	National Centre for Youth Substance Use Research, The University of Queensland, Brisbane, Australia				0000-0002-1824-1000	No conflict of interest	N/A
Michael	Patrick	Schaub	Swiss Research Institute for Public Health and Addiction, associated to University of Zurich, Zurich, Switzerland				0000-0002-8375-4005	No conflict of interest	N/A
Mauro		Schiavella	B-ASC Bicocca Applied Statistics Center, University of Milano-Bicocca, Milan, Italy	Seafrog Technology S.r.l., Milan, Italy			0009-0005-0691-2371	Mauro Schiavella is one of the founding partners of a company that produces responsible gambling systems (Seafrog Technology S.r.l., Milan, Italy).	Mauro Schiavella For the participation in this research project I have received no specific grant from any funding agency in the public, commercial, or not-for-profit sectors.
Adriano		Schimmenti	Department of Human and Social Sciences, UKE - Kore University of Enna,Enna, Italy				0000-0001-5181-2648	No conflict of interest	Adriano Schimmenti received funding for gambling research by the Regione Siciliana (Fondo per il Gioco d'azzardo patologico)
Casper		Schmidt	Department of Communication and Psychology, Aalborg University, Aalborg, Denmark				0000-0001-6290-2040	No conflict of interest	N/A
Guillaume		Sescousse	Lyon Neuroscience Research Centre — INSERM U1028 — CNRS UMR5292, PSYR2 Team, University of Lyon, Lyon, France				0000-0002-8556-3755	No conflict of interest	N/A
Serge		Sévigny	Département des fondements et pratiques en éducation, Université Laval, Québec, (QC), Canada				0000-0002-5361-8432	No conflict of interest	N/A
Howard	Jeffrey	Shaffer	Division on Addiction, Cambridge Health Alliance, Malden, MA, USA	Harvard Medical School, Department of Psychiatry, Boston, MA, USA			0000-0001-7003-5362	No conflict of interest	N/A

Steve		Sharman	National Addiction Centre, Institute of Psychiatry, Psychology and Neuroscience, King's College London, London, UK				0000-0001-9816-7981	Steve Sharman is a member of the Advisory Board for Safer Gambling (ABSG), Co-Executive Chair of the Current Advances in Gambling Research Conference (CAGR), an Executive Committee member of the Academic Forum for the Study of Gambling (AFSG) and a Co-Chair of the AFSG Outreach Committee	Steve Sharman is currently funded by a fellowship from UKRI, and is further funded by grants from the NIHR, and Greo.
Jing		Shi	Health and Social Sciences, Singapore Institute of Technology, Singapore				0000-0001-7156-8128	No conflict of interest	N/A
Steven	D.	Shirk	Research & Development, VA Bedford Healthcare Center, Bedford, MA, USA	Department of Psychiatry and Population and Quantitative Health Sciences, University of Massachusetts Chan Medical School, Worcester, MA, USA			0000-0003-1905-1505	No conflict of interest	Steven Shirk has received funding from the International Center for Responsible Gaming. This institute had no influence on the answers given for this project.
Lucia		Sideli	Department of Human Science, LUMSA University Rome, Rome, Italy				0000-0001-6124-6897	No conflict of interest	N/A
Olivier		Simon	Centre Du Jeu Excessif, Addiction Medicine, Lausanne University Hospital, Lausanne, Switzerland	Collège romand de médecine de l'addiction, Lausanne, Switzerland			0000-0002-9216-5886	No conflict of interest	Olivier Simon is an employee of the public university hospital (State of Vaud / Switzerland)
Pawel		Sleczka	Faculty of Psychology, DHGS Deutsche Hochschule für Gesundheit und Sport, Berlin, Germany				0000-0002-2393-7539	No conflict of interest	N/A
Ryuhei		So	Okayama Psychiatric Medical Center, Okayama, Japan	Departments of Health Promotion and Human Behavior, Kyoto University Graduate School of Medicine, Kyoto, Japan	CureApp, Inc., Tokyo, Japan		0000-0002-9838-350X	Ryuhei So has received speaker's honoraria from Otsuka Pharmaceutical Co., Ltd., Nippon Shinyaku Co., Ltd., and Takeda Pharmaceutical Co., Ltd., outside the submitted work. Ryuhei So also reports an employment position at CureApp Inc., which develops software as medical devices.	Ryuhei So has received research grants from the Japan Society for the Promotion of Science (JSPS); Ministry of Health, Labor and Welfare, Japan; Japan Agency for Medical Research and Development; Osake-no-Kagaku Foundation; The Mental Health Okamoto Memorial Foundation; and Kobayashi Magobe Memorial Medical Foundation.
Rhys	Michael Geoffrey	Stevens	University of Lethbridge, Lethbridge, Alberta, Canada				0000-0002-4985-1987	No conflict of interest	N/A
Trevor		Steward	University of Melbourne, Faculty of Medicine, Dentistry and Health Sciences Melbourne School of Psychological Sciences, Parkville, Australia				0000-0003-3116-8175	No conflict of interest	Trevor Steward is supported by a NHMRC/MRFF Investigator Grant (MRF1193736), a BBRF Young Investigator Grant, and a University of Melbourne McKenzie Fellowship

Mythily		Subramaniam	Research Division, Institute of Mental Health, Singapore	Saw Swee Hock School of Public Health, Singapore			0000-0003-4530-1096	No conflict of interest	N/A
Thomas	B.	Swanton	The University of Sydney, School of Psychology, Brain & Mind Centre, Gambling Treatment & Research Clinic, Sydney, NSW, Australia				0000-0002-5111-2534	Thomas B. Swanton has received a PhD scholarship through the NSW Government's Gambling Research Capacity Grants program, funded by the NSW Responsible Gambling Fund, and supported by the NSW Office of Responsible Gambling. He has been awarded student travel grants by the National Association for Gambling Studies.	N/A
André		Syvertsen	Department of Psychosocial Science, University of Bergen, Norway	Norwegian Competence Center for Gambling and Gaming Research, University of Bergen, Norway,			0000-0002-3881-3758	No conflict of interest	N/A
Nassim		Tabri	Department of Psychology, Carleton University, Ottawa, Ontario, Canada				0000-0002-7085-9350	No conflict of interest	N/A
Hidehiko		Takahashi	Department of Psychiatry and Behavioral Sciences, Graduate School of Medical and Dental Sciences, Institute of Science Tokyo, Tokyo, Japan				0000-0002-5102-1982	No conflict of interest	N/A
Stefano		Tamburin	Department of Neurosciences, Biomedicine and Movement Sciences, University of Verona, Verona, Italy				0000-0002-1561-2187	No conflict of interest	N/A
So Kum	Catheri ne	Tang	Hong Kong Shue Yan Univeristy, Hong Kong, China				0000-0002-7577-4372	No conflict of interest	N/A
Éric	R.	Thériault	Department of Psychology, Cape Breton University, Sydney, N.S., Canada				0000-0002-0460-6670	No conflict of interest	Éric R. Thériault is currently conducting research funded by Gambling Awareness Nova Scotia - Administered by Research Nova Scotia.
Shane	Andre w	Thomas	Institute of Health and Wellbeing, Federation University, Ballarat, Vic, Australia				0000-0003-3116-6022	Shane Andree Thomas receives support for his research from Australian and Victorian government funding bodies. He does not receive support from the gambling industry.	N/A
Kristine	Rømer	Thomsen	Department of Psychology and Behavioural Sciences, Centre for Alcohol and Drug Research, Aarhus University, Denmark				0000-0003-3612-5529	No conflict of interest	N/A

Alexander		Tomei	Centre for Excessive Gambling, Addiction Medicine, Department of Psychiatry, Lausanne University Hospital and University of Lausanne, Lausanne, Switzerland				0000-0001-9949-3498	No conflict of interest	N/A
Kwok Kit		Tong	Department of Psychology, University of Macau, Macau, China				0000-0001-8594-9300	No conflict of interest	N/A
Joan		Trujols	Unitat de Conductes Addictives, Servei de Psiquiatria, Hospital de la Santa Creu i Sant Pau, Institut d'Investigacions Biomèdiques Sant Pau (IIB Sant Pau), Barcelona, Spain	Centro de Investigación Médica en Red de Salud Mental (CIBERSAM), Instituto de Salud Carlos III, Madrid, Spain			0000-0003-0396-7105	No conflict of interest	N/A
Samson		Tse	Department of Social Work and Social Administration, Faculty of Social Sciences, The University of Hong Kong, Hong Kong, China				0000-0001-9003-1086	No conflict of interest	N/A
Kosuke		Tsurumi	Department of Psychiatry, Kyoto University Graduate School of Medicine, Kyoto, Japan				0000-0001-5051-6978	No conflict of interest	N/A
Catherine		Tulloch	Central Queensland University, Experimental Gambling Research Laboratory, School of Health, Medical and Applied Sciences, Sydney, Australia				0000-0002-2842-5110	No conflict of interest	Catherine Tulloch has received funding from Central Queensland University, the New South Wales Office of Responsible Gambling, the Victorian Responsible Gambling Foundation and Gambling Research Australia.
Richard		Tunney	School of Psychology, Aston University, Birmingham, England				0000-0003-4673-757X	No conflict of interest	N/A
Eduardo		Valenciano-Mendoza	Atenció i Investigació de Socioaddiccions (AIS), Barcelona, Spain	Psychoneurobiology of Eating and Addictive Behaviours Group, Neurosciences Program, Bellvitge Biomedical Research Institute (IDIBELL), Barcelona, Spain			0000-0002-1690-5109	Eduardo Valenciano-Mendoza has received consultancy honoraria from Novo Nordisk	N/A
Mark		van der Maas	Center for Gambling Studies, Rutgers University, New Brunswick, New York, USA				0000-0001-9606-0187	No conflict of interest	Mark van der Maas is currently receiving support from the International Center for Responsible Gaming

Thilo		Van Eimeren	Department of Neurology, University Hospital Cologne, University of Cologne, Cologne, Germany	Department of Nuclear Medicine, University Hospital Cologne, University of Cologne, Cologne, Germany			0000-0002-6951-2325	Thilo Van Eimeren received honoraria for consulting or in advisory roles from Lundbeck Foundation, Lundbeck Pharma, Orion Pharma, GT Gain Therapeutics SA, EISAI GmbH, ICON PLC, Inserm France. He received non-monetary research support from Life Molecular Imaging, Lilly Germany, Neuroclues. He owns tock ownership: IBM, Microsoft, NVIDIA.	Thilo Van Eimeren received monetary research support from Brandau-Leibach Stiftung, DFG (German Research Foundation), JPND (EU Joint Programme for Neurodegenerative Diseases), EBC (European Brain Council).
William		Van Gordon	School of Psychology, University of Derby, Derby, UK				0000-0002-5648-3043	No conflict of interest	N/A
Ruth	J.	Van Holst	Department of Psychiatry, AmsterdamUMC - University of Amsterdam, Amsterdam, the Netherlands	Centre for Urban Mental Health, University of Amsterdam, Amsterdam, the Netherlands			0000-0002-1184-9355	Ruth J. Van Holst is an Associate Editor of the Journal of Behavioral Addictions and a Regional Assistant Editor for the journal International Gambling Studies	N/A
Tim		Van Timmeren	Department of Social, Health and Organizational Psychology, Utrecht University, Utrecht, The Netherlands				0000-0003-0282-8269	No conflict of interest	N/A
Patrizia		Velotti	Department of Dynamic and Clinical Psychology, and Health Studies, Sapienza University of Rome				0000-0002-1933-8314	No conflict of interest	N/A
Cristina		Vintró-Alcaraz	Department of Mental Health, Hospital de Mataró, Consorci Sanitari del Maresme, Mataró, Spain				0000-0001-9453-8810	No conflict of interest	N/A
Rachel		Volberg	School of Public Health and Health Sciences, University of Massachusetts Amherst, Amherst, MA, USA	Gemini Research, Inc., Northampton, MA, USA			0000-0003-2959-0748	No conflict of interest	N/A
Kristin	M.	von Ranson	Department of Psychology, University of Calgary, Calgary, Alberta, Canada	Mathison Centre for Research and Education/Hotchkiss Brain Institute, University of Calgary, Calgary, Alberta, Canada	O'Brien Institute for Public Health, University of Calgary, Calgary, Alberta, Canada	Alberta Children's Hospital Research Institute, University of Calgary, Calgary, Alberta, Canada	0000-0001-6023-7948	No conflict of interest	N/A
Kathrin		Weidacker	School of Psychology, Swansea University, Swansea, United Kingdom				0000-0002-5742-6016	No conflict of interest	N/A
James	P.	Whelan	TN Institute for Gambling Education & Research, Department of				0000-0002-2245-6450	No conflict of interest	James P. Whelan has received funding by the TN Department of

			Psychology, Memphis TN USA						Mental Health and Substance Abuse Services
Seth		Whiting	Louisiana State University Shreveport, Shreveport, Louisiana, USA				0000-0001-6451-9010	No conflict of interest	N/A
Łukasz		Wieczorek	Institute of Psychiatry and Neurology, Department of Studies on Alcoholism and Drug Dependence, Warsaw, Poland				0000-0002-9636-3695	No conflict of interest	N/A
Robert		Williams	University of Lethbridge Lethbridge, Alberta, Canada				0000-0002-9558-9588	No conflict of interest	N/A
Ken	C.	Winters	Oregon Research Institute, St. Paul, MN location, OR, USA				0000-0002-5406-2923	No conflict of interest	N/A
Michael	J. A.	Wohl	Carleton University, Ottawa, Canada				0000-0001-6945-5562	Michael J. A. Wohl has received research funding from Alberta Gambling Research Institute (Canada), British Columbia Lottery Corporation (Canada), Carleton University (Canada), Gambling Research Exchange Ontario (Canada), Manitoba Gambling Research Program (Canada), International Center for Responsible Gaming (US), Ontario Lottery and Gaming (Canada), and Ontario Ministry of Health and Long-Term Care (Canada). MW has received speaker/travel honorarium from Alberta Liquor Gaming Commission (Canada), National Association for Gambling Studies (Australia), International Center for Responsible Gaming (US), and Massachusetts Council on Compulsive Gambling (US), New York Council of Problem Gambling (US); Problem Gambling Ohio Network (US); Safe Foundation (US); The Star Entertainment Group (Australia). He has received fees for academic services from Atlantic Lottery and Gaming Corporation (Canada), Gambling Research Exchange (Canada), National Center for Responsible Gaming (US), New South Wales Government (Australia), Nova Scotia Gaming Corporation (Canada), Manitoba Gambling Research Program (Canada), Massachusetts Gambling Commission (US), and Ontario Lottery and	Michael J. A. Wohl is currently receiving support from the International Center for Responsible Gaming and the Massachusetts Gaming Commission

								Gaming (Canada). MW has also received consulting fees from Alberta Liquor Gaming Commission (Canada), Aristocrat Gaming (US); Atlantic Lottery and Gaming Corporation (Canada), GamRes (Canada), Massachusetts Gaming Commission (US), National Council on Problem Gambling (Singapore), Nova Scotia Gaming Corporation (Canada), and Ontario Lottery and Gaming (Canada).	
Patrick	D.	Worhunsky	Department of Psychiatry, Yale University School of Medicine, New Haven, CT, USA				0000-0001-9629-1428	No conflict of interest	N/A
Leon Y.		Xiao	School of Creative Media, City University of Hong Kong, China	beClaws.org, London, United Kingdom			0000-0003-0709-0777	Leon Y. Xiao has provided paid consultancy and research services for (i) Public Group International Ltd (t/a PUBLIC) (Companies House number: 10608507), commissioned by the UK Department for Culture, Media and Sport (DCMS) to conduct independent research on understanding player experiences of loot box protections (October 2024 – May 2025); (ii) the Council of Europe International Cooperation Group on Drugs and Addiction (the Pompidou Group) on a project concerning the risks of online gambling and gaming to young people co-funded by the	Leon Y. Xiao is supported by a Presidential Assistant Professors Scheme Start-Up Research Grant awarded by the City University of Hong Kong [香港城市大學] (March 2025). Until November 2024, Leon Y. Xiao was supported by a PhD Fellowship funded by the IT University of Copenhagen (IT-Universitetet i København), which is publicly funded by the Kingdom of Denmark (Kongeriget Danmark).

								European Union via the Technical Support Instrument and implemented by the Council of Europe, in cooperation with the European Commission (December 2024 – May 2025); and (iii) the Institute of Public Health on a report concerning the advertising of harmful products to children funded by the Irish Department of Health and intended for its Online Health Taskforce (July 2025). L.Y.X. was employed by LiveMe, then a subsidiary of Cheetah Mobile (NYSE: CMCM), as an in-house counsel intern from July to August 2019 in Beijing, China. L.Y.X. was not involved with the monetisation of video games by Cheetah Mobile or its subsidiaries. L.Y.X. undertook a brief period of voluntary work experience at Wiggin LLP (Solicitors Regulation Authority number: 420659) in London, England, in August 2022. L.Y.X. has contributed to research projects enabled by data access provided by the video game industry, specifically Unity Technologies (NYSE:U) (October 2022 – August 2023). L.Y.X. has been invited to provide advice to the UK Department for Digital, Culture, Media and Sport and its successor (the Department for Culture, Media and Sport; DCMS) on the technical working group for loot boxes and the Video Games Research Framework. L.Y.X. was the (co-)recipient of three Academic Forum for the Study of Gambling (AFSG) postgraduate research support grants (March 2022, January 2023, and July 2024) and a minor exploratory research grant (May 2024) derived from ‘regulatory settlements applied for socially responsible purposes’ received by the UK Gambling Commission and administered by Gambling Research Exchange Ontario (GREO) and its successor (Greo Evidence Insights; Greo). L.Y.X. accepted funding to publish open-access academic papers from GREO and the AFSG that was received by the UK Gambling Commission as above (October, November, and	

								December 2022, November 2023, and May 2024). L.Y.X. was the recipient of an Elite Research Travel Grant 2024 [EliteForsk-rejsestipendium 2024] awarded by the Agency for Higher Education and Science of the Danish Ministry of Higher Education and Science [Uddannelses-og Forskningsstyrelsen under Uddannelses-og Forskningsministeriet] (February 2024). L.Y.X. has accepted conference travel and attendance grants from the Socio-Legal Studies Association (February 2022 and February 2023); the Current Advances in Gambling Research Conference Organising Committee with support from GREO (February 2022); the International Relations Office of The Jagiellonian University (Uniwersytet Jagielloński), the Polish National Agency for Academic Exchange (NAWA; Narodowa Agencja Wymiany Akademickiej), and the Republic of Poland (Rzeczpospolita Polska) with co-financing from the European Social Fund of the European Commission of the European Union under the Knowledge Education Development Operational Programme (May 2022); the Society for the Study of Addiction (November 2022, March 2023, and November 2024); the organisers of the 13th Nordic SNSUS (Stiftelsen Nordiska Sällskapet för Upplysning om Spelberoende; the Nordic Society Foundation for Information about Problem Gambling) Conference, which received gambling industry sponsorship (January 2023); the MiSK Foundation (Prince Mohammed bin Salman bin Abdulaziz Foundation) (November 2023); and the UK Gambling Commission (March 2024). L.Y.X. has received honoraria from the Center for Ludomani for contributing parent guides about mobile games for Tjekspillet.dk, which was funded by the Danish Ministry of Health's gambling addiction pool (Sundhedsministeriets Ludomanipulje) (March and	

								December 2023), the Fundació Pública Tecnocampus Mataró-Maresme (TecnoCampus Mataró-Maresme Foundation) for a guest lecture (November 2023), the Young Men's Christian Association (YMCA) of Greater Toronto Youth Gambling Awareness Program for a presentation, which was funded by the Government of Ontario, Canada (March 2024), Lunds universitet (Lund University) for the right to translate parent guides about mobile games into Swedish for Kollaspelet.se, which was funded by Mediamyndigheten (the Swedish Agency for the Media) and Barnahus Stockholm (December 2024); Shenkar College of Engineering, Design and Art for a guest lecture (December 2024); and DiGRA Korea and the Game-n-Science Institute [게임과학연구원] under the Game Culture Foundation [게임문화재단] under the Ministry of Culture, Sports and Tourism of South Korea [문화체육관광부] for participating in an academic research survey (January 2025). L.Y.X. received royalties by virtue of the copyright subsisting in some of his publications from the Authors' Licensing and Collecting Society (ALCS) (Companies House number: 01310636) (March 2023, 2024, & 2025). A full gifts and hospitality register-equivalent for L.Y.X. is available at: https://www.leonxiao.com/about/gifts-and-hospitality-register. The up-to-date version of L.Y.X.'s conflict-of-interest statement is available at: https://www.leonxiao.com/about/conflict-of-interest.	
Kengo		Yokomitsu	School of Psychological Sciences, University of Human Environments, Matsuyama, Ehime, Japan				0000-0002-3242-7776	Kengo Yokomitsu received personal fees from a for-profit company promoting integrated resorts in Japan and abroad, domestic tobacco companies, and CureApp Inc.	N/A

Murat		Yucel	QIMR-Berghofer, Brisbane, QLD, Australia	BrainPark, Monash University, Melbourne, ViC, Australia			0000-0002-4705-452X	No conflict of interest	Murat Yucel receives funding from: government funding bodies such as the NHMRC, Australian Research Council (ARC), Australian Defence Science and Technology (DST), the Department of Industry, Innovation and Science (DIIS), the National Institutes of Health (NIH, USA); philanthropic donations from the David Winston Turner Endowment Fund, Wilson Foundation; sponsored Investigator-Initiated trials including Incannex Healthcare Ltd; and payments in relation to court-, expert witness-, and/or expert review-reports. These funding sources had no role in the data analysis, presentation, or interpretation and write-up of the data. Murat Yucel also sits on the Advisory Boards of: Centre of The Urban Mental Health, University of Amsterdam; Monash Biomedical Imaging Centre; and Enosis Therapeutics.
Martin	Henry	Zack	Molecular Brain Sciences Research Department, Centre for Addiction and Mental Health, Toronto, Canada	Department of Pharmacology & Toxicology, University of Toronto, Toronto, Canada			0000-0001-9992-3160	No conflict of interest	N/A
Meng Xuan		Zhang	Department of Medical Humanities, School of Humanities, Southeast University, Nanjing, Jiangsu, China				0000-0003-1648-732X	No conflict of interest	N/A
Alexander		Zink	Department of Dermatology and Allergy, School of Medicine, Technical University of Munich, Munich, Germany	Division of Dermatology and Venereology, Department of Medicine Solna, Karolinska Institutet, Stockholm, Sweden			0000-0001-9313-6588	No conflict of interest	N/A
Francesca		Zoratto	Centre for Behavioural Sciences and Mental Health, Istituto Superiore di Sanità, Rome, Italy				0000-0002-3626-8928	No conflict of interest	N/A
